# Genome-wide comparative analysis of RNA-binding Glycine-rich protein family genes between *Gossypium arboreum* and *Gossypium raimondii*

**DOI:** 10.1371/journal.pone.0218938

**Published:** 2019-06-26

**Authors:** Wencui Yang, Min Yu, Changsong Zou, Cairui Lu, Daoqian Yu, Hailiang Cheng, Pengfei Jiang, Xiaoxu Feng, Youping Zhang, Qiaolian Wang, Hong Zhang, Guoli Song, Zhuqing Zhou

**Affiliations:** 1 Laboratory of Cell Biology, College of Life Science and Technology, Huazhong Agricultural University, Wuhan, China; 2 State Key Laboratory of Cotton Biology, Cotton Research Institute, Chinese Academy of Agricultural Sciences, Anyang, China; 3 Key Laboratory of Plant Stress Biology, State Key Laboratory of Cotton Biology, School of Life Sciences, Henan University, Kaifeng, China; National Botanical Research Institute CSIR, INDIA

## Abstract

*RB-GR*P (RNA-binding Glycine-rich protein gene) family belongs to the fourth subfamily of the GRP (Glycine-rich protein gene) superfamily, which plays a great role in plant growth and development, as well as in abiotic stresses response, while it has not been identified in cotton. Here, we identified 37 and 32 *RB-GRPs* from two cotton species (G*ossypium arboreum* and G*ossypium raimondii*, respectively), which were divided into four distinct subfamilies based on the presence of additional motifs and the arrangement of the glycine repeats. The distribution of *RB-GRPs* was nonrandom and uneven among the chromosomes both in two cotton species. The expansion of *RB-GRP* gene family between two cultivars was mainly attributed to segmental and tandem duplication events indicated by synteny analysis, and the tandem duplicated genes were mapped into homologous collinear blocks, indicated that they shared a common ancestral gene in both species. Furthermore, most *RB-GRPs* in two cotton species undergone stronger negative selective pressure by evolutionary analysis of *RB-GRP* orthologous genes. Meanwhile, *RB-GRPs* participated in different abiotic stresses (Abscisic acid, salt and Polyethylene glycol) responses and tissues at different developmental stages between two cotton species were showed by gene expression analysis. This research would provide insight into the evolution and function of the *RB-GRPs* in *Gossypium* species.

## Introduction

Glycine-rich proteins (GRPs) are a group of proteins mainly constituted with glycine, which was first discovered in *Petunia* and *Cucurbita* [1]. GRPs could govern gene expression at transcriptional or post-transcriptional levels of RNA during plant development. Moreover, they also participate in the post-transcriptional regulation triggered by different environmental stresses, such as water, temperature, light or low-oxygen stress [2]. In plants, GRPs were grouped into four classes (class I, II, III and IV) by the presence of additional motifs and the arrangement of the glycine repeats [3]. Here, Class IV *GRPs* will be elaborated in this study which are also known as RNA-binding *GRPs* (*RB-GRPs*), which contain either an RNA-recognition motif (RRM) or a cold-shock domain (CSD), in addition to the CCHC (cysteine-cysteine-histidine-cysteine) zinc-fingers and the glycine-rich domain [4]. *RB-GRPs* could also be subdivided into four subgroups, IVa (which contains an RRM), IVb (one RRM and a CCHC zinc-finger), IVc (a CSD and two or more zinc-fingers), and IVd (two RRMs) based on the diversity of domain arrangements [4].

In the past decade, numerous *RB-GRP* encoding genes have been isolated and identified among different plants sequentially. For instance, eight and six *RB-GRPs* have been discovered in *Arabidopsis* and rice by genome analysis [5, 6], 23 *RB-GRPs* in maize [7]. *RB-GRPs* play great important roles in plant growth, development, and stress resistance, as the presence of RRM, CSD, or CCHC domains [8]. *AtGRP2* and *AtGRP7* could enhance the tolerance of cold and freezing in *Arabidopsis* [9]. Eight *ZmRB-GRPs* significantly responded to cold, salt and ABA stresses, and they also be involved in other physiological processes of maize under multiple stresses according to expression profile analysis [7]. *RB-GRP2* could promote the germination of *Arabidopsis* seed and growth of seedlings [10]. The transcription of *MhGR-RBP1* were remarkably inhibited after exogenous JA (jasmonic acid) and ABA treatment [11], while expressions of three *RB-GRPs* (*GRP2*, *GRP4* and *GRP7*) increased significantly during plant acclimation to cold [12]. CSDPs (cold shock domain proteins), contain one cold shock domain at N-terminus and glycine-rich regions interspersed with CCHC-type zinc finger at the C-terminal, play a significant role in plant growth and development as well as resistant to cold stress [13, 14]. *CSDP1* from *Arabidopsis thaliana* could complement the cold sensitivity of *BX*04 mutant *Escherichia* coli, and resulted in better survival rate than control at low temperature, which implied that *CSDP1* could exhibit RNA chaperone activity during the cold adaptation process [15].

Cotton (*Gossypium* spp.) is an important economic crop that produces the most important natural resources for the textile industry [16], while it also conducts as a model plant for study of polyploidy cell elongation and cell wall synthesis in scientific research [17]. Researches on genome of diploid cotton were increasing in recent years [17, 18, 19, 20], provides help for the extensive identification of gene family. As is known to all, all tetraploid cotton species were derived from interspecific hybridization between A (*G*.*arboreum*) and D-genome species (*G*.*raimondii*) [21]. Thus, *G*.*arboreum* (A_2_) and *G*.*raimondii* (D_5_) were assumed to be the donor material tetraploid cotton. However, the function of *RB-GRPs* in cotton remains unknown. Here, a systematic study of *RB-GRP* gene family in *G*.*arboreum* and *G*.*raimondii* to identify the characterization and phylogenetic relationships between the two species was conducted and predicted. The development of cotton fiber consists of four stages, including fiber initiation, cell elongation, secondary wall deposition and maturation [22], while the *RB-GRPs* whether participate in development of cotton is still unknown, thus, expressions of *RB-GRPs* in fiber and seed during different developmental stages were discussed in this study. Moreover, the expression patterns of *RB-GRPs* under different stress conditions were also surveyed. It would offer a diploid reference for the analysis of cotton agronomic traits, such as the quality of fiber and resistance to stress.

## Materials and methods

### Data resource

The *G*.*arboreum* gene information was downloaded from CGP (http://cgp.genomics.org.cn), and the *G*.*raimondii* genome database was obtained from phytozome (https://phytozome.jgi.doe.gov/pz/portal.html#!info?alias=Org_Graimondii). *Arabidopsis thaliana* genomic data was downloaded from TAIR10 (http://www.arabidopsis.org). *Theobroma cacao* data was downloaded from Ensemblplants (http://plants.ensembl.org). Maize (*Zea mays* L.) genomic data was downloaded from Gramene (http://maizesequence.org). RRM, RRM-related and CSD hmm files (PF00076, PF04059, PF08777, PF10378, PF10598, PF13893, PF14259, PF00313) were retrieved from pfam (http://pfam.sanger.ac.uk/).

### Identification and classification of *RB-GRPs* in *G*.*aboreum* and *G*.*raimondii*

In the draft genome of *G*.*aboreum*, *RB-GRPs* were identified using Hidden Markov Model (HMM) profile corresponding to the Pfam RRM family (PF00076, PF04059, PF08777, PF10378, PF10598, PF13893, PF14259) and CSD family PF00313 through the HMMER 3.0 package with the E-value<1×10^−4^ as threshold. From the selected protein sequence screened through RRM domain and CSD domain, the *RBP*s containing high content of glycine residues (more than 50% residues within any 20-animo acid peptide are glycine) were obtained using perl script. Then, the *RB-GRPs* were confirmed within the database SMART (http://samart.embl-heidelberg.de/) and INTERPRO (http://www.ebi.ac.uk/interpro/) according to their conserved domain architecture. The *RB-GRPs* were divided into different subgroups, Sector allotment was based on their conserved motif composition as described previously. Molecular weight (MW), theoretical isoelectric point (pI), and size of the RB-GRPs were investigated with the online tool ExPASy (http://expasy.org/tools/). Subcellular locations were predicted by software WoLF PSORT (http://wolfpsort.org/).

### Chromosome distribution of *RB-GRPs*

The positions of the *RB-GRP* genes were physically mapped to the 13 chromosomes in each genome with GFF file downloaded from http://cgp.genomics.org.cn, respectively. After that, Mapchart was used to draw the physical maps of *RB-GRPs* on chromosomes with SVG module.

### Multiple sequence alignment and phylogenetic analyses of *RB-GRP* genes

To explore the evolution relationship of *RB-GRP* genes, conserved domains among five species have been extracted using perl script and aligned according to the “hmmalign” module by HMMER V3.0 programmer, and the files containing conserved domain sequences should be converted into a format that MEGA 5.0 can recognize. Then the resulting sequences were used to construct a phylogenetic tree using the N-J method in MEGA 5.0 with the random seed of phylogeny test, poisson correction and pairwise deletion option parameters enable. A bootstrap test with 1,000 replicates was tested to obtain the reliability of the trees, and only a test value higher than 50% in the clades was selected for the conserve tree.

#### Exon/intro structure analysis and motif prediction of *RB-GRPs* between *G*.*arboreum* and *G*.*raimondii*

The existing gff files of *G*.*arboreum* and *G*.*raimondii* were downloaded from http://cgp.genomics.org.cn, and then extracted the files contained candidate *RB-GRP* genes to analyze the exon/intro structures by GSDS (http://gsds.cbi.pku.edu.cn). Conserved motifs and zinc finger structures of the selected protein sequences were confirmed by SMART database (http://smart.embl-heidelberg.de/) and INTEPRO (http://www.ebi.ac.uk/interpro/scan.html), respectively.

### Detection of collinear tandem arrays

A tandem array at an ancestral locus which termed collinear tandem array, may imply positional gene family expansion. In this study, BLASTP was provided for detection of collinear tandem arrays of *RB-GRP* genes between *G*.*arboreum* and *G*.*raimondii*. In any BLASTP hit, the two genes are relabeled as ‘tandem duplicates’ if they have a difference of gene rank = 1.

### Identification and non-synonymous/synonymous substitution (Ka/Ks) ratios of orthologous gene pairs of *BP-GRP* genes between *G*.*aboreum* and *G*.*raimondii*

A two-step method was used to obtain the orthologous gene pairs of *BP-GRP* genes to detect the evolutionary relationship between different cotton species. Firstly, MCscanX was employed to identify the orthologous regions between *G*.*arboreum* and *G*.*raimondii*. Secondly, orthologous gene pairs of *RB-GRP* genes were then extracted according to orthologous regions containing *RB-GRP* genes with small Ks value. PAML package was used to calculate the orthologous rate of Ka and Ks to characterize the collinear genes between *G*.*arboreum* and *G*.*raimondii*.

### Plant materials and growth condition

*G*.*arboreum* and *G*.*raimondii* were grown in soil mixture in a climate-controlled greenhouse (16 h light/8 h dark at 30°C). 250 mM NaCl, 100 mg/L ABA and 10% PEG (Polyethylene glycol) were treated after the expansion of the first true leaf to induce salt stress, hormone stress and drought stress, respectively. For each induction treatment, we collected leaf samples at five time points (0 as control, 6, 12, 24 and 48 h). To analyze the expressions of *RB-GRPs* in different tissues, plants were tagged on the day of flowering (0 DPA), fiber was separated from 50 plants at 0 DPA (ovule), 3 DPA, 6 DPA, 10 DPA and 15 DPA, and seed at 10 DPA, 20 DPA, 30 DPA and 40 DPA. And then immediately frozen in liquid nitrogen and stored at -80°C freezer for RNA extraction. Three biological replicates were conducted for each sample.

### RNA isolation and qPCR analysis

Total RNA of all the collected samples were extracted using the RNAprep Pure Plant kit (Aidlab, Beijing, China). A total of 2 μg of RNA was used as the template, and the first-strand cDNAs were synthesized using the Takara Reverse Transcription System (TaKaRa, Shuzo, Otsu, Japan). The gene expressions of all *RB-GRP* genes were detected by the 170-8792iCycler iQ Calibration Kit qPCR (Quantitative Real-time polymerase chain reaction) system (Bio-Rad, USA). SYBR Green Real-time PCR Master Mix (Toyobo) was used to perform the reaction. The details of the protocol were as follows: (Step 1) initial denaturation step of 30 s at 95°C, (Step 2) 40 cycles of 5 s at 95°C, 34 s at 60°C and (Step 3) melting curve analysis, and the comparative Ct (2^-ΔΔCt^) method was used to calculate gene expression levels. The β-actin gene was chosen as the reference gene. The primer sequences are shown in S1 Table. Specificity of primers used in this study was verified by subcloning the generated amplicons using the TOPO TA Cloning Kit (Thermo Fischer Scientific, Reinach, Switzerland), and then using them for sequencing (data not shown). Gradient dilution of validated plasmids was then used to construct a standard curve. Amplification efficiency of primer pairs of all genes we detected were no less than 98%. Each experiment was repeated three times.

### RNA-sequencing analysis

Total RNA was extracted using the RNAprep Pure Plant kit (Aidlab, Beijing, China).CA, USA) from different cotton tissues during different development stages. The RNA samples were sent to the Beijing Berrygenomics for sequencing on an Illumina HiSeq2000 sequencing platform. The DEGseq package was used for identifying genes differentially expressed between paired samples pairings, and P-values were adjusted according to the Benjamini and Hochberg method [23].

## Results

### Identification and classification of *BP-GRP* genes in *G*.*aboreum* and *G*.*raimonii*

Until now, 23 and 8 glycine-rich RNA-binding protein genes were identified in the genomes of *Zea mays* and *Arabidopsis thaliana*, respectively [5, 7]. 434 and 405 non-redundant RNA-binding protein (RBP)-coding genes were identified by the HMM profile from the Pfam database in the genome assemblies of *G*.*arboreum* and *G*.*raimondii*, respectively. 50 and 47 RNA-binding glycine-rich protein genes were then selected according to presence of (Gly)n-X repeats in the 434 and 405 *RB-GRPs*. The protein sequences of above candidate genes were then confirmed within the SMART database (http://smart.embl-heidelberg.de/) and BLASTP according to the conserved domains of their own. Finally, 37 and 32 *RB-GRP* genes were selected from *G*.*arboreum* and *G*.*raimondii* (S1 and S2 Figs). Meanwhile, we have identified 13 and 15 glycine-rich RNA-binding protein genes in the genomes of *Arabidopsis thaliana* and *Theobroma cacao* using the same method (Table 1). Then we categorized these *RB-GRP* encoding genes into four subtribes (IVa, IVb, IVc and IVd) according to domain motif consistent with previous principles of classification (Table 1) [4]. According to Table 1, numbers of Class IVa in genomes of five different plant species were all bigger than any other subtribes, followed by the Class IVd. In addition, the numbers of RB-GRP genes in two cotton species were bigger than other plant species. *GaRB-GRP*1 to *GaRB-GRP*37 and *GrRB-GRP*1 to *GrRB-GRP*33 were ordered according to Tables 2 and 3.

**Table 1:** Statistics of predicted *RB-GRP* genes in sequenced plant species.

Class	Domain origination	Ga	Gr	Tc	Zm	At
a	RRM	14	11	6	6	8
b	RRM-C_**2**_HC	4	6	0	6	3
c	CSD-C_**2**_HC*-*C_**2**_HC	7	6	0	2	2
d	RRM- RRM	12	9	9	9	0
Total		37	32	15	23	13

**Note:** Ga, *G*. *arboreum*; Gr, *G*. *raimondii*; Tc, *Theobroma cacao*; Zm, *Zea mays*; At, *Arabidopsis thaliana*.

**Table 2:** The information of *RB-GRP* genes from *G*.*arboreum*.

Gene name	Gene identifier	Genomics position	Domain	Class	Size(aa)	Mw(kDa)	*pI*	SL
*GaRB-GRP1*	*Cotton_A_18641*	Chr12: 121743510–121745456	RRM	IVa	1947	154.40	4.99	Nucl
*GaRB-GRP2*	*Cotton_A_35587*	Chr10: 63405763–63408849	RRM	IVa	714	75.96	5.54	Nucl
*GaRB-GRP3*	*Cotton_A_13382*	Chr06: 99467873–99469938	RRM	IVa	620	67.81	5.61	Nucl
*GaRB-GRP4*	*Cotton_A_19121*	Chr01: 83119516–83121726	RRM	IVa	204	21.70	4.33	Chlo
*GaRB-GRP5*	*Cotton_A_25290*	Chr05: 58953175–58954484	RRM	IVa	143	15.19	7.89	Chlo
*GaRB-GRP6*	*Cotton_A_18157*	Chr04: 46366311–46368236	RRM	IVa	266	27.34	4.59	Nucl
*GaRB-GRP7*	*Cotton_A_19718*	Chr13: 63500417–63501198	RRM	IVa	168	17.00	7.84	Nucl
*GaRB-GRP8*	*Cotton_A_30104*	Chr02: 39345844–39347785	RRM	IVa	277	27.90	4.83	Chlo
*GaRB-GRP9*	*Cotton_A_00739*	Chr05: 7739595–7740294	RRM	IVa	150	15.30	7.82	Nucl
*GaRB-GRP10*	*Cotton_A_10822*	Chr06: 66607573–66608404	RRM	IVa	180	17.49	5.58	Nucl
*GaRB-GRP11*	*Cotton_A_38392*	Chr13: 49886706–49887861	RRM	IVa	162	17.29	8.56	Nucl
*GaRB-GRP12*	*Cotton_A_29989*	Chr01: 145186127–145187256	RRM	IVa	160	17.17	8.97	Nucl
*GaRB-GRP13*	*Cotton_A_22958*	Chr08: 124343839–124346641	RRM	IVa	285	31.47	11.11	Nucl
*GaRB-GRP14*	*Cotton_A_23360*	Chr13: 67682667–67685020	RRM	IVa	284	32.16	10.85	Nucl
*GaRB-GRP15*	*Cotton_A_35063*	Chr08: 87050125–87051448	RRM-C_2_HC	IVb	172	19.42	11.07	Nucl
*GaRB-GRP16*	*Cotton_A_09110*	Chr13: 49095156–49096462	RRM-C_2_HC	IVb	176	19.71	10.85	Nucl
*GaRB-GRP17*	*Cotton_A_18468*	Chr08: 44375570–44376091	CSD-C_2_HC-C_2_HC	IVc	173	18.08	8.56	Nucl
*GaRB-GRP18*	*Cotton_A_00105*	Chr07: 38178647–38182879	RRM-RRM	IVd	643	67.27	5.08	Nucl
*GaRB-GRP19*	*Cotton_A_28705*	Chr13: 60595149–60601311	RRM-RRM	IVd	779	81.93	5.03	Nucl
*GaRB-GRP20*	*Cotton_A_22297*	Chr10: 79030956–79041224	RRM-RRM	IVd	993	105.25	4.91	Nucl
*GaRB-GRP21*	*Cotton_A_20124*	Chr07: 115880136–115883234	RRM-RRM	IVd	470	47.84	6.31	Nucl
*GaRB-GRP22*	*Cotton_A_31097*	Chr11: 99209223–99211584	RRM-RRM	IVd	371	38.65	7.11	Nucl
*GaRB-GRP23*	*Cotton_A_22861*	Chr05: 42988292–42990818	RRM-RRM	IVd	499	50.65	8.53	Nucl
*GaRB-GRP24*	*Cotton_A_22864*	Chr05: 42941988–42944656	RRM-RRM	IVd	510	51.72	7.63	Nucl
*GaRB-GRP25*	*Cotton_A_26974*	Chr01: 51641998–51643980	RRM-RRM	IVd	337	35.46	8.92	Nucl
*GaRB-GRP26*	*Cotton_A_03266*	Chr03: 27599804–27601949	RRM-RRM	IVd	336	35.66	7.79	Cyto
*GaRB-GRP27*	*Cotton_A_40291*	Chr01: 93291477–93293503	RRM-RRM	IVd	341	35.93	9.01	Cyto
*GaRB-GRP28*	*Cotton_A_15644*	Chr04: 137194101–137197237	RRM-RRM	IVd	479	52.83	4.83	Nucle
*GaRB-GRP29*	*Cotton_A_25535*	Chr12: 23068372–23071485	RRM-RRM	IVd	472	52.08	4.97	Nucl
*GaRB-GRP30*	*Cotton_A_16121*	Chr10: 23944428–23944985	CSD-C_2_HC	IVc	185	19.05	9.08	Nucl
*GaRB-GRP31*	*Cotton_A_00241*	Chr07: 39155418–39155876	CSD-C_2_HC	IVc	152	15.43	6.81	Nucl
*GaRB-GRP32*	*Cotton_A_34922*	Chr07: 12787980–12788510	CSD-C_2_HC	IVc	176	17.09	6.81	Nucl
*GaRB-GRP33*	*Cotton_A_39551*	Chr02: 97043251–97043775	CSD-C_2_HC	IVc	174	16.79	6.29	Nucl
*GaRB-GRP34*	*Cotton_A_00650*	Chr05: 8445845–8446339	CSD-C_2_HC	IVc	164	16.31	6.29	Nucl
*GaRB-GRP35*	*Cotton_A_11378*	Chr06: 77256797–77257528	CSD-C_2_HC-C_2_HC-C_2_HC	IVc	243	25.53	7.00	Nucl
*GaRB-GRP36*	*Cotton_A_35374*	Chr08: 33322492–33326283	RRM-RanBP_2_-RanBP_2_	IVb	383	42.30	8.25	Nucl
*GaRB-GRP37*	*Cotton_A_38093*	Chr06: 57591767–57595780	RRM-RanBP_2_-RanBP_2_	IVb	388	43.01	8.86	Nucl

**Note:** Mw, Molecular weight; SL, Subcellular localization. The same is below.

**Table 3:** The information of *RB-GRP* genes from *G*.*raimondii*.

Gene name	Gene identifier	Genomics position	Domain	Class	Size(aa)	Mw(kDa)	*pI*	SL
*GrRB-GRP1*	*Gorai*.*012G173800*	Chr12: 34341214–34347064	RRM	IVa	648	69.15	5.75	Nucl
*GrRB-GRP2*	*Gorai*.*007G038400*	Chr07: 2655660–26596	RRM	IVa	718	76.46	5.49	Nucl
*GrRB-GRP3*	*Gorai*.*008G195400*	Chr08:48022470–48026788	RRM	IVa	710	75.35	5.64	Nucl
*GrRB-GRP4*	*Gorai*.*001G077000*	Chr01:7977562–7980895	RRM	IVa	294	30.59	4.99	Chlo
*GrRB-GRP5*	*Gorai*.*005G028100*	Chr05: 2458681–2460986	RRM	IVa	143	15.22	7.90	Nucl
*GrRB-GRP6*	*Gorai*.*003G101300*	Chr03:31097257–31100241	RRM	IVa	257	26.78	4.63	Nucl
*GrRB-GRP7*	*Gorai*.*005G243900*	Chr05: 62432126–62433175	RRM	IVa	150	15.28	6.31	Nucl
*GrRB-GRP8*	*Gorai*.*007G039400*	Chr07: 2741633–2744829	RRM	IVa	278	28.06	4.76	Chlo
*GrRB-GRP9*	*Gorai*.*013G036800*	Chr13: 2909216–2910436	RRM	IVa	169	17.09	7.84	Nucl
*GrRB-GRP10*	*Gorai*.*010G201600*	Chr10: 56416759–56420481	RRM	IVb	286	31.64	11.04	Nucl
*GrRB-GRP11*	*Gorai*.*011G262000*	Chr11: 59312836–59316206	RRM	IVa	302	34.04	10.72	Nucl
*GrRB-GRP12*	*Gorai*.*010G121100*	Chr10: 24510307–24513576	RRM-C_2_HC	IVb	183	20.75	11.34	Nucl
*GrRB-GRP13*	*Gorai*.*010G245400*	Chr10: 61433489–61436975	RRM-C_2_HC	IVb	240	27.23	11.30	Chlo
*GrRB-GRP14*	*Gorai*.*001G074700*	Chr01: 7640275–7644569	RRM-C_2_HC	IVb	205	22.19	9.25	Nucl
*GrRB-GRP15*	*Gorai*.*008G193200*	Chr08: 47725290–47729452	RRM-C_2_HC	IVb	209	22.66	9.07	Nucl
*GrRB-GRP16*	*Gorai*.*010G139100*	Chr10: 33197125–33204400	RRM-C_2_HC	IVb	208	22.45	8.33	Nucl
*GrRB-GRP17*	*Gorai*.*010G048300*	Chr10: 5276099–5276620	CSD-C_2_HC-C_2_HC	IVc	173	18.26	7.60	Nucl
*GrRB-GRP18*	*Gorai*.*002G015800*	Chr02: 1020186–1025274	RRM-RRM	IVd	639	66.81	5.03	Nucl
*GrRB-GRP19*	*Gorai*.*009G372200*	Chr09: 50402594–50408320	RRM-RRM	IVd	432	44.19	7.77	Nucl
*GrRB-GRP20*	*Gorai*.*002G063400*	Chr02: 7420662–7424240	RRM-RRM	IVd	438	44.70	7.73	Nucl
*GrRB-GRP21*	*Gorai*.*006G034100*	Chr06: 9011445–9014344	RRM-RRM	IVd	371	38.62	7.11	Nucl
*GrRB-GRP22*	*Gorai*.*005G053000*	Chr05: 5244825–5248496	RRM-RRM	IVd	458	46.29	6.53	Nucl
*GrRB-GRP23*	*Gorai*.*005G052700*	Chr05: 5195162–5198813	RRM-RRM	IVd	457	46.00	6.53	Nucl
*GrRB-GRP24*	*Gorai*.*001G098600*	Chr01: 11089609–11093105	RRM-RRM	IVd	337	35.42	8.92	Cyto
*GrRB-GRP25*	*Gorai*.*004G061200*	Chr04: 6042369–6045834	RRM-RRM	IVd	337	35.53	7.74	Cyto
*GrRB-GRP26*	*Gorai*.*007G112300*	Chr07: 8678759–8683729	RRM-RRM	IVd	479	52.79	4.86	Nucl
*GrRB-GRP27*	*Gorai*.*009G243500*	Chr09: 19496296–19497353	CSD-C_2_HC	IVc	185	19.00	9.23	Nucl
*GrRB-GRP28*	*Gorai*.*005G253200*	Chr05: 63097845–63098505	CSD-C_2_HC	IVc	116	11.95	5.06	Chlo
*GrRB-GRP29*	*Gorai*.*002G195000*	Chr02: 52786495–52787903	CSD-C_2_HC	IVc	175	16.94	6.81	Nucl
*GrRB-GRP30*	*Gorai*.*002G195100*	Chr02: 52798613–52802367	CSD-C_2_HC	IVc	177	16.99	6.81	Nucl
*GrRB-GRP31*	*Gorai*.*002G003500*	Chr02: 195345–196248	CSD-C_2_HC	IVc	169	17.39	8.15	Chlo
*GrRB-GRP32*	*Gorai*.*002G167100*	Chr02: 41459084–41464174	RRM-RanBP_2_-RanBP_2_	IVb	388	42.95	8.00	Nucl

### Chromosome location of *RB-GRPs* between *G*.*arboreum* and *G*.*raimondii*

**Fig 1:**
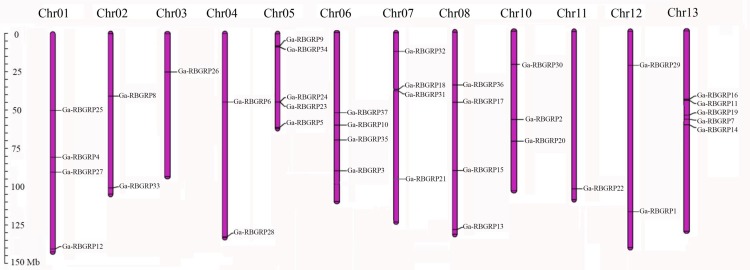
Chromosomal localization of *RB-GRP* genes in *G*.*arboreum*. The scale on the left was in megabases (Mb).

The 37 *GaRB-GRP* genes were located on the 13 *G*.*arboreum* chromosomes (Fig 1). Normally, the number of *GaRB-GRP* genes on each chromosome varied widely. Chromosome 5 and chromosome 13 have a maximum of five *GaRB-GRP* genes, respectively. Four *GaRB-GRP* genes on chromosome 1, 6, 7 and 8, followed by on chromosome 10 which three members were found. Chromosome 2, and 4 contained two genes each, whereas each only single *GaRB-GRP* gene was localized on chromosome 3, 11 and 12 (Fig 1). Obviously, they were distributed unevenly among 13 chromosomes, except for no *GaRB-GRP* gene was found on chromosome 9 (Fig 1). Four pairs of *GaRB-GRP*s were linked on the same chromosome. The rest genes were found as singletons on chromosomes (Fig 1).

**Fig 2:**
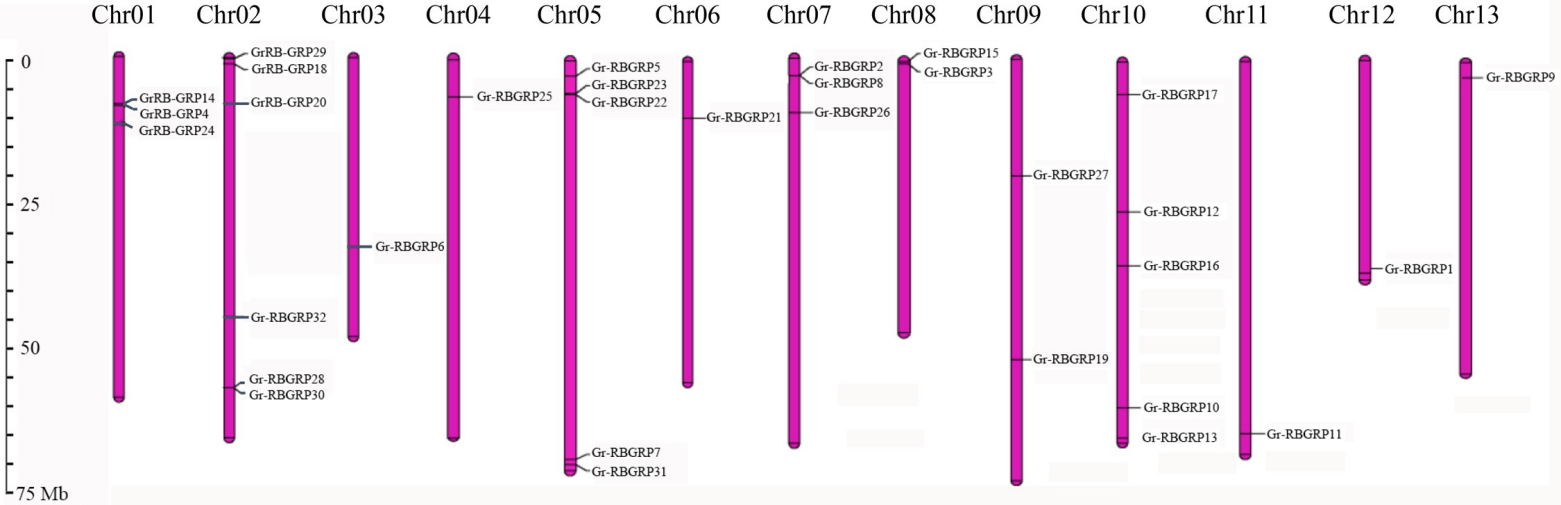
Chromosomal localization of *RB-GRP* genes in *G*.*raimondii*. The scale on the left was in megabases (Mb).

Like the case in *G*.*arboreum*, the 32 *GrRB-GRP* genes distributed unevenly across the 13 chromosomes in *G*.*raimondii* (Fig 2). Chromosomes 2 had a maximum of six *GrRB-GRP* genes, five *GrRB-GRP* genes each on chromosome 5 and 10, respectively, three on chromosome 1 and 7, two on chromosome 9 and 8, one each distributed on the other four chromosomes, respectively (Fig 2). Five pairs of *GrRB-GRP*s in *G*.*raimondii* were linked on the same chromosome (Fig 2).

### Phylogenetic analysis of *RB-GRP* gene family

**Fig 3:**
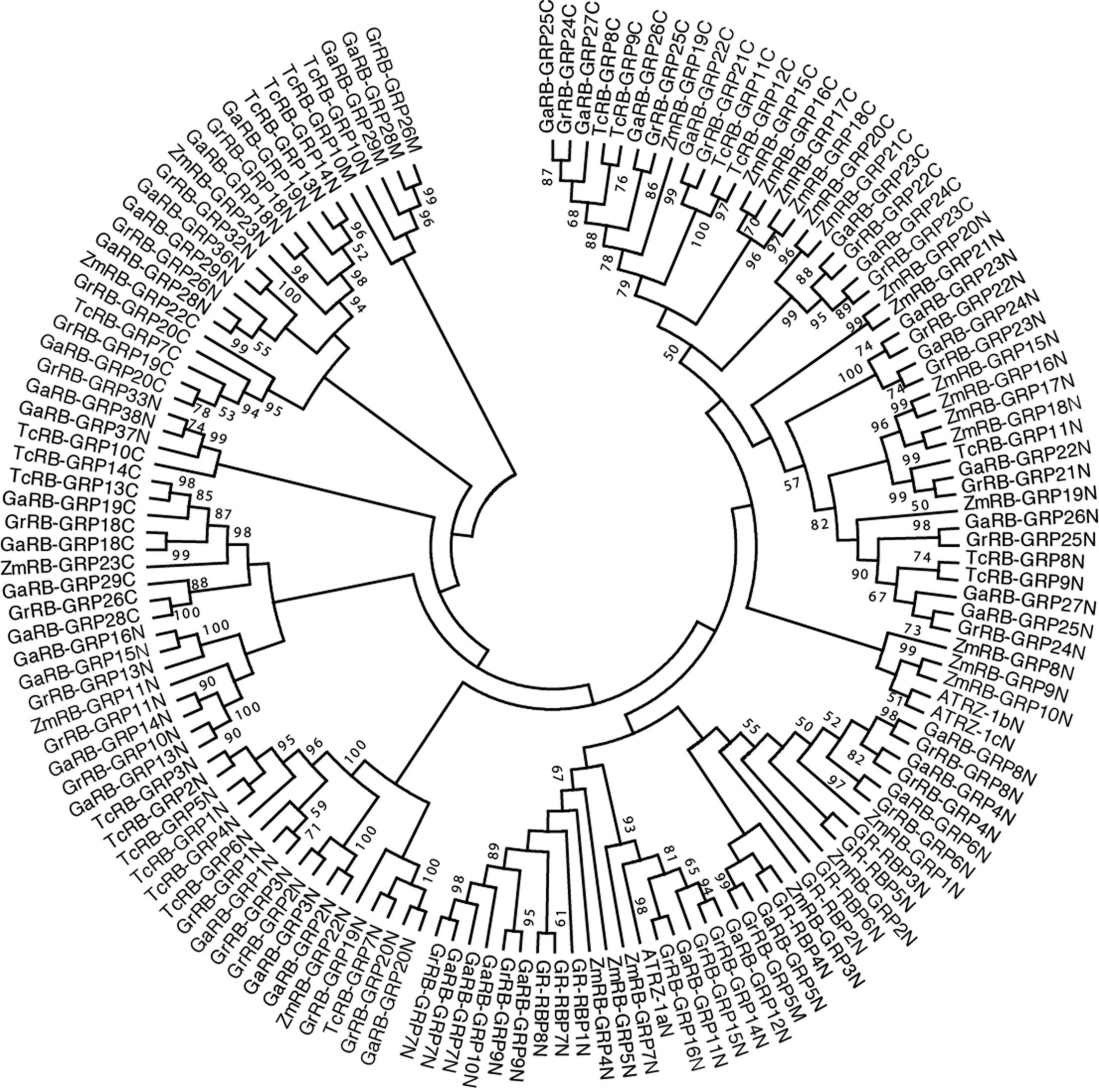
Phylogenetic relationships of *RB-GRP* genes from *G*.*arboreum*, *G*.*raimondii*, *Arabidopsis thaliana*, *Theobroma cacao* and *Zea mays*. The unrooted phylogentic tree was constructed using MEGA 4.0 by Neighbor-Joining method and the bootstrap test was performed with 1000 replicates. Percentage bootstrap scores of > 50% were displayed.

To investigate the molecular evolution of *RB-GRP* gene family comprehensively and systematically, all the putative *RB-GRPs* (with protein conserved domains) from two cotton species, as well as the *RB-GRPs* from *Arabidopsis thaliana*, *Theobroma cacao* and *Zea mays*, were aligned to generate an unrooted phylogenetic tree separately with Neiboring-Joining method. According to the position among the protein sequence, the domain sequence naming scheme was added a suffix N, M and C behind the original sequence name, such as *GaRB-GRP37* N, *GaRB-GRP37* M and *GaRB-GRP37* C. There are two domains (RRM and CSD) existed in *RB-GRP*s, while the RRM-type *RB-GRPs* accounted for the majority. Thus, the RRM-type phylogenetic tree of RB-GRP proteins from *G*.*raimondii* or *G*.*arboreum*, *Arabidopsis thaliana*, *Theobroma cacao* and *Zea mays* was established (Fig 3). It suggested that most of RRM-type *RB-GRPs* of five species were divided into two subgroups according to the position of RRM domain (C-terminal or N-terminal), expect for two *GaRB-GRPs* (*GaRB-GRP28* and *GaRB-GRP29*), two *GrRB-GRP*s (*GrRB-GRP5* and *GrRB-GRP26*) and *TcRB-GRP 10*.

### Gene structure of *GaRB-GRPs* and *GrRB-GRPs*

**Fig 4:**
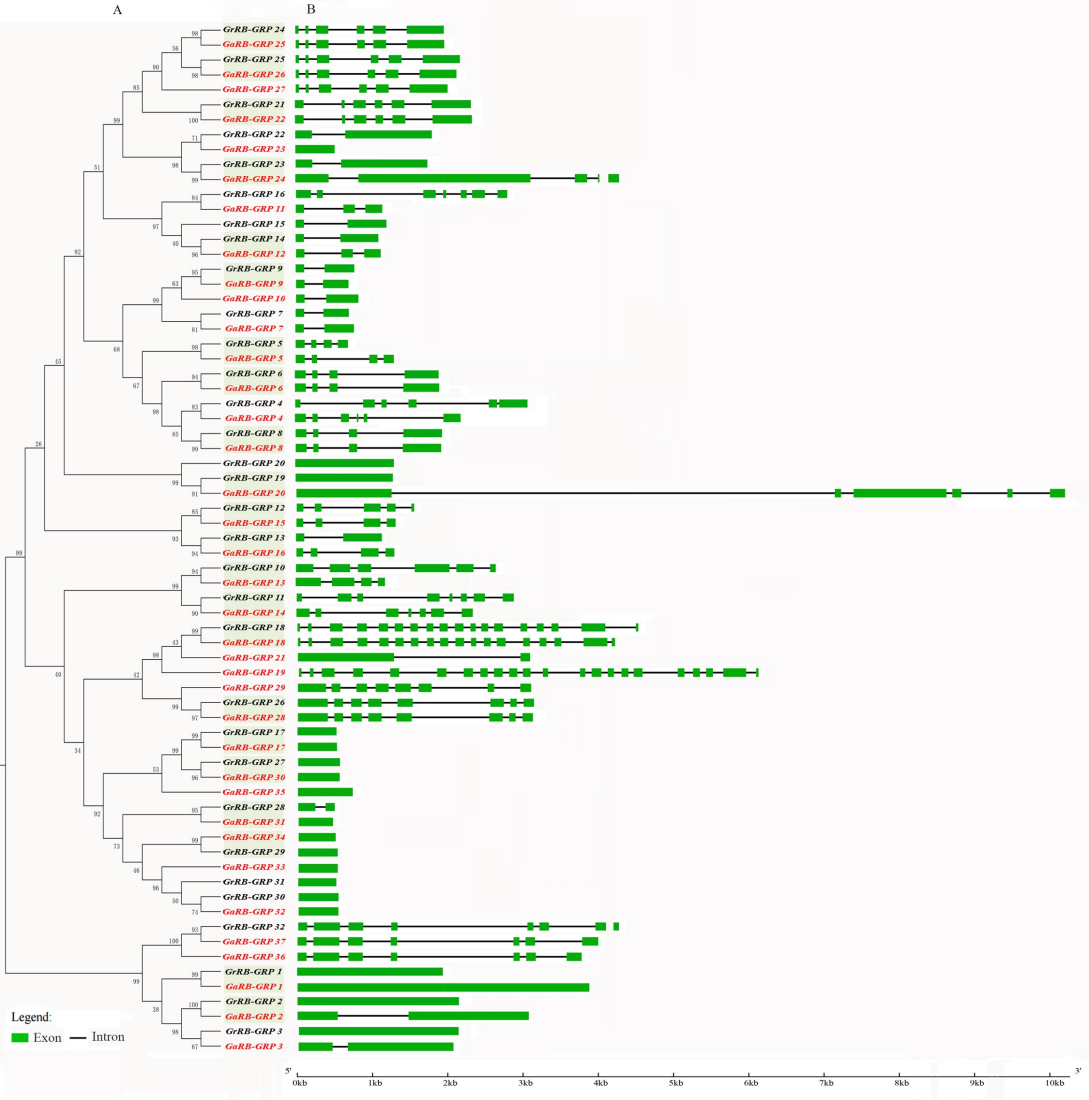
Phylogenetic relationships and gene structure of Ga*RB-GRP*s and Gr*RB-GRP*s. A: The phylogenetic tree of all *RB-GRP* genes in *G*.*arboreum* and *G*.*raimondii*. The names of *GaRB-GRP*s and Gr*RB-GRP*s were marked with red and black, respectively. Gene names in gray background shown orthologous pairs. B: The exon-intron structure of all *RB-GRP* genes from *G*.*arboreum* and *G*.*raimondii*. Exons were represented by green boxes and introns by gray lines.

To investigate the possible structural evolution of *RB-GRP* gene family in the two diploid cotton species, the gene structures of *GaRB-GRPs* and *GrRB-GRPs* were compared separately. In general, the exon/intron organizations of *RB-GRPs* were consistent with the phylogenetic subfamilies showed in Fig 4. In general, the exon/intron organizations of *RB-GRPs* were consistent with the phylogenetic subfamilies showed in Fig 4, and the gene structures were conserved within the same group. Most members of *RB-GRPs* possessed two or more exons, *GaRB-GRP19* had 22 exons, which is the maximum in all the *RB-GRP* genes, followed by *GaRB-GRP18* and *GrRB-GRP18*. The gene structures of *RB-GRP* orthologous pairs were almost identical with only minor differences, with the exception of *GrRB-GRP23*/*GaRB-GRP24*, *GrRB-GRP13*/*GaRB-GRP16*, *GrRB-GRP10*/*GaRB-GRP13*, *GrRB-GRP19*/*GaRB-GRP20* and *GrRB-GRP2*/*GaRB-GRP2*.

### Whole genome collinearity analysis of *RB-GRPs* between *G*.*arboreum* and *G*.*raimondii*

**Fig 5:**
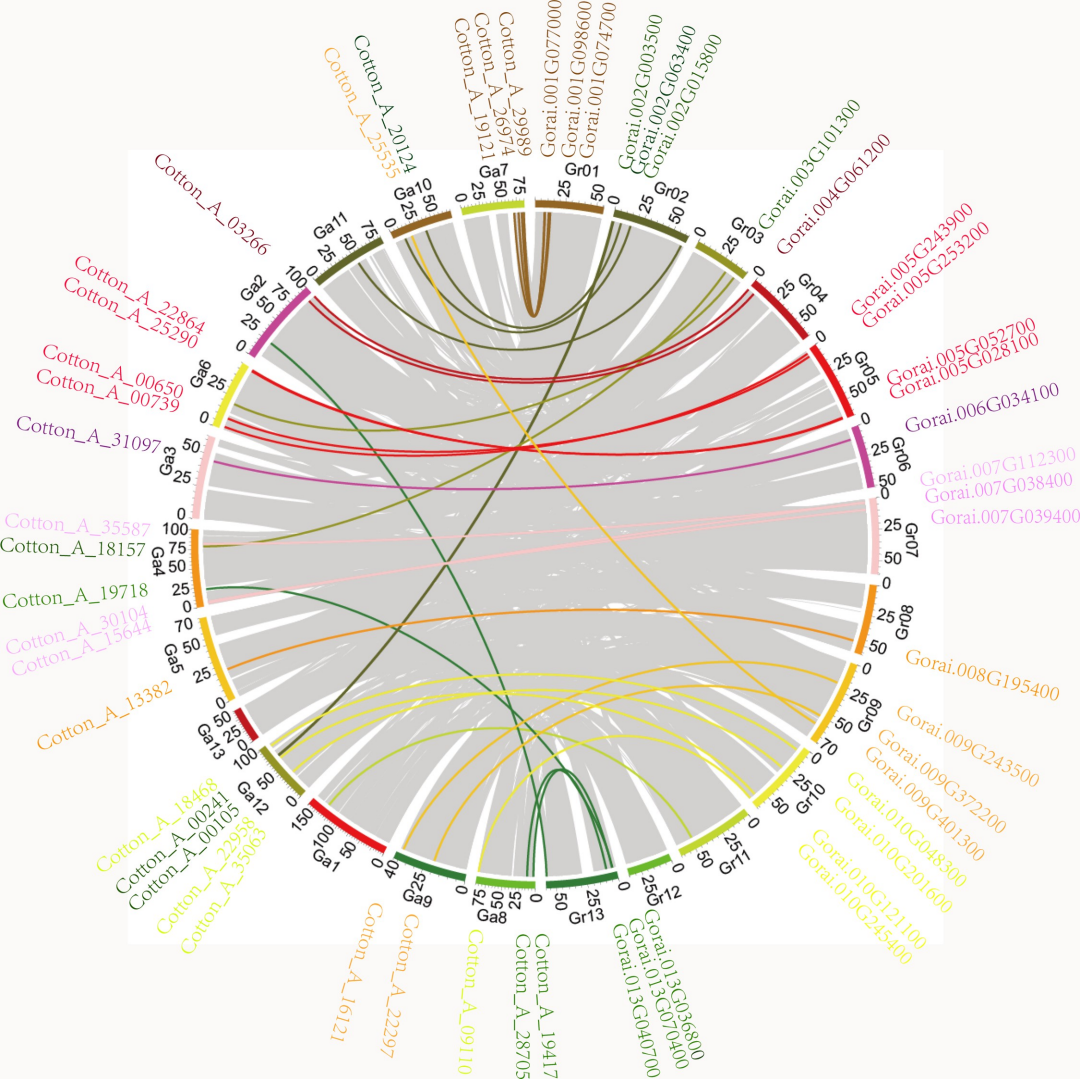
Collinear gene pairs of orthologous *RB-GRP* genes between *G*.*arboreum* and *G*.*raimondii*. The duplicated gene pairs and orthologous relationships between the genomes are represented by Circos figure.

The *RB-GRP* gene family in *G*.*arboreum* and *G*.*raimondii* have established a close relationship of collinear and synteny for each other (Fig 5), exploited by Circos software. The analysis of collinear blocks of two cotton species indicated that the large-scale syntenies contained 27 *GaRB-GRPs*, 26 *GrRB-GRPs* and one gene (*Gorai*.*009G401300*) in *G*.*raimondii* genome that was identified as not *RB-GRP* gene found to share synteny with *G*.*arboreum*. 10 *RB-GRPs* were single *G*.*arboreum*-to-*G*.*raimondii* orthologs, which indicated these genes should have been in the genome of the last common ancestor of *G*.*arboreum* and *G*.*raimondii*. While the rest genes showed one-to-more or more-to-one correspondence, for example, twelve cases that two *G*.*arboreum* genes corresponded to one *G*.*raimondii* genes, thirteen cases that one *G*.*arboreum* gene corresponded to multiple *G*.*raimondii* genes.

### Expansion pattern of *RB-GRP* genes in *G*.*arboreum* and *G*.*raimondii*

Detection of collinear orthologs is important for understanding gene evolution. Segment and tandem duplications are the main mechanism resulting in gene family expansion. Distribution of paralogs could be used to analysis the potential duplications and the evolution of *RB-GRPs* between *G*.*arboreum* and *G*.*raimondii*. Sixteen paralogs *GaRB-GRPs* and twelve *GrRB-GRPs* were detected based on the bootstrap value in the phylogenetic analysis, and all of them randomly distributed on chromosomes of *G*.*arboreum* and *G*.*raimondii*, which indicated that segmental duplication is predominant during *RB-GRP* gene family expansion. Meanwhile, *Cotton_A_22861*/*Cotton_A_22864*, *Gorai*.*005G053000*/*Gorai*.*005G052700* and *Gorai*.*002G019510*/*Gorai*.*002G019500* were six tandem duplication genes, and *Cotton_A_22861*/*Gorai*.*005G052700* belonged to the orthologous pair of single *G*.*arboreum*-to-*G*.*raimondii* gene correspondence. It illustrated expansion of *RB-GRP* gene family was also associated with tandem duplication event, and the expansion pattern of *RB-GRP*s in *G*.*arboreum* is consistent with *G*.*raimondii*.

### Selective pressure analysis of orthologous gene pairs for *RB-GRP* genes

To investigate the selective constrains on *RB-GRP* genes, the non-synonymous (Ka) to synonymous (Ks) substitution ratio for orthologous gene pairs for *RB-GRP* genes were calculated. As is known to all that the ratio (Ka/Ks) indicated neutral mutation when Ka equals to Ks, negative (purifying) selection when ka is less than Ks, and positive (diversifying) selection when Ka exceeds Ks [24]. In this study, 20 orthologous gene pairs in the *RB-GRP* gene family of *G*.*arboreum* and *G*.*raimondii* were investigated (S3 Fig). For RRM-type, the mean Ka/Ks ratio of all of orthologous genes was 0.154 between *G*.*arboreum* and *G*.*raimondii*, with most of them being even<0.3, which suggested that they had experienced strong purifying selection pressure (S3 Fig). 3 out of 4 orthologous gene pairs had undergone purifying selection pressure, and only one pair (*Cotton_A_18468*-*Gorai*.*010G048300*) with a ratio>1 were found in CSD-type (S3 Fig). Those observations reflected that the functions of *RB-GRPs* in different cottons did not diverge much during subsequent evolution. And the purifying selection might contribute mainly to the maintenance of function in *RB-GRP* gene family.

### Expression analysis of *RB-GRP*s during fiber and seed development between *G*.*arboreum* and *G*.*raimondii*

**Fig 6:**
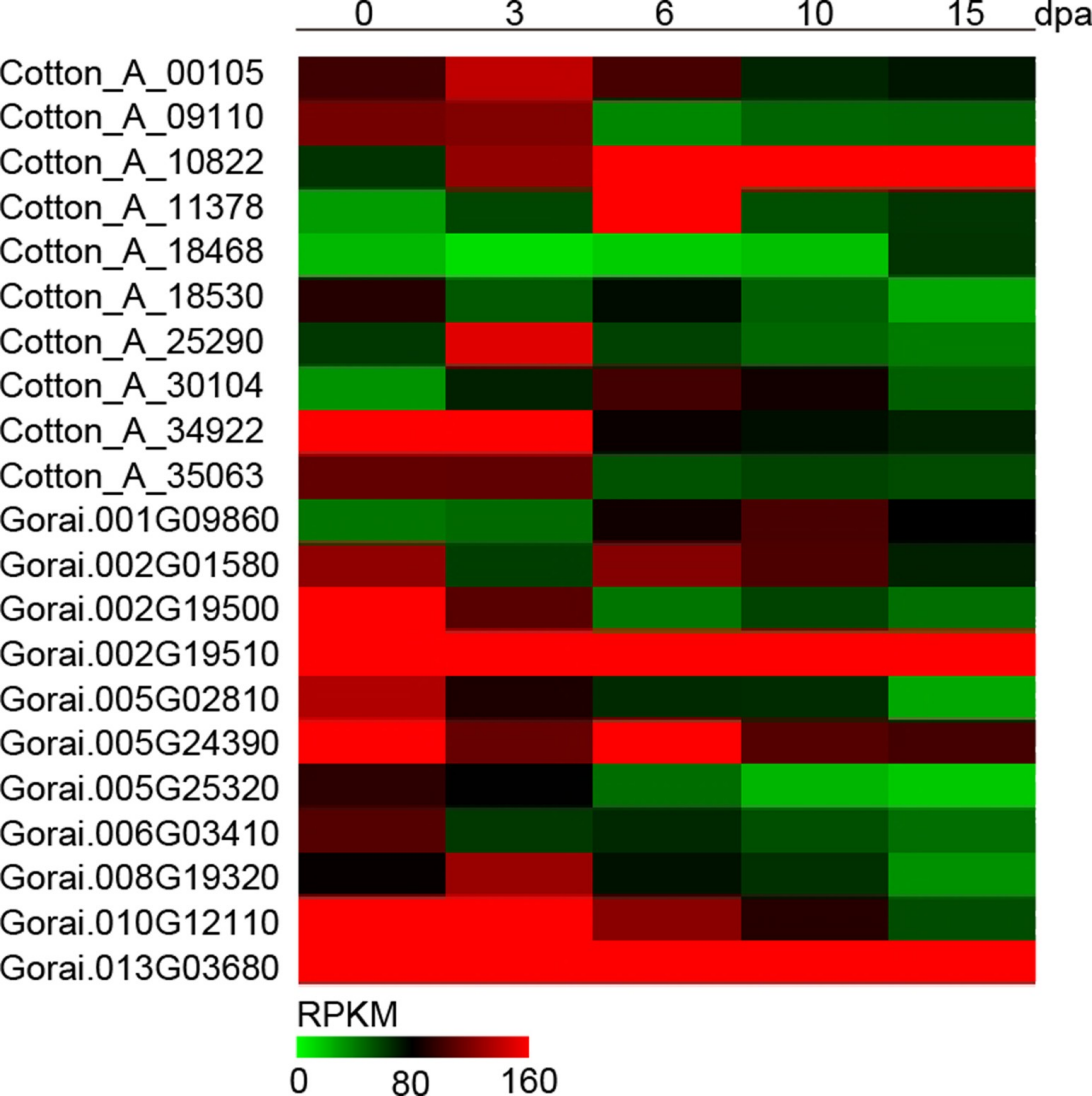
Heatmap of the differentially expressed *RB-GRP* genes in fibers during different developmental stages between *G*.*arboreum* and *G*.*raimondii*.

**Fig 7:**
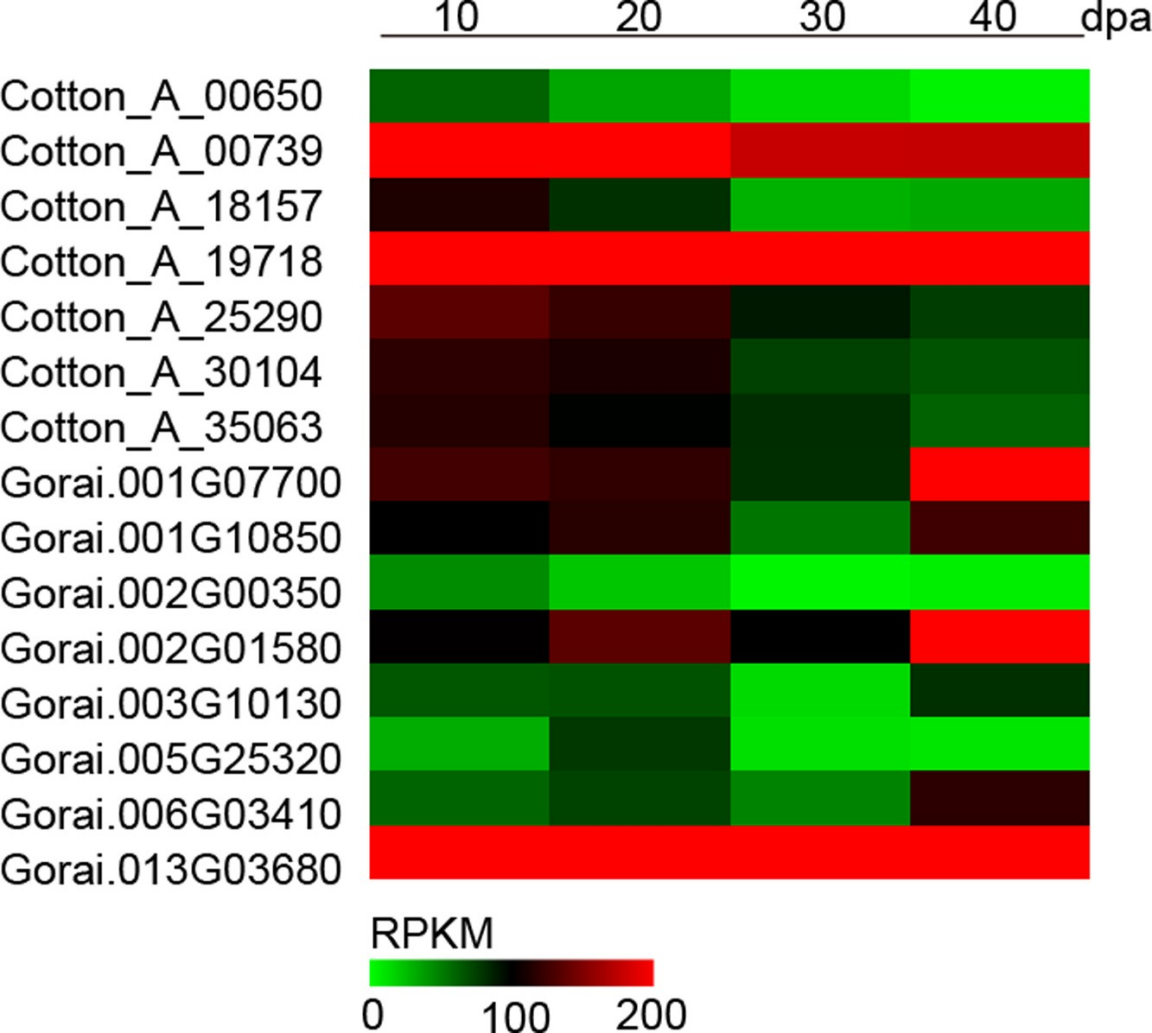
Heatmap of the differentially expressed *RB-GRP* genes in seeds during different developmental stages between *G*.*arboreum* and *G*.*raimondii*.

**Fig 8:**
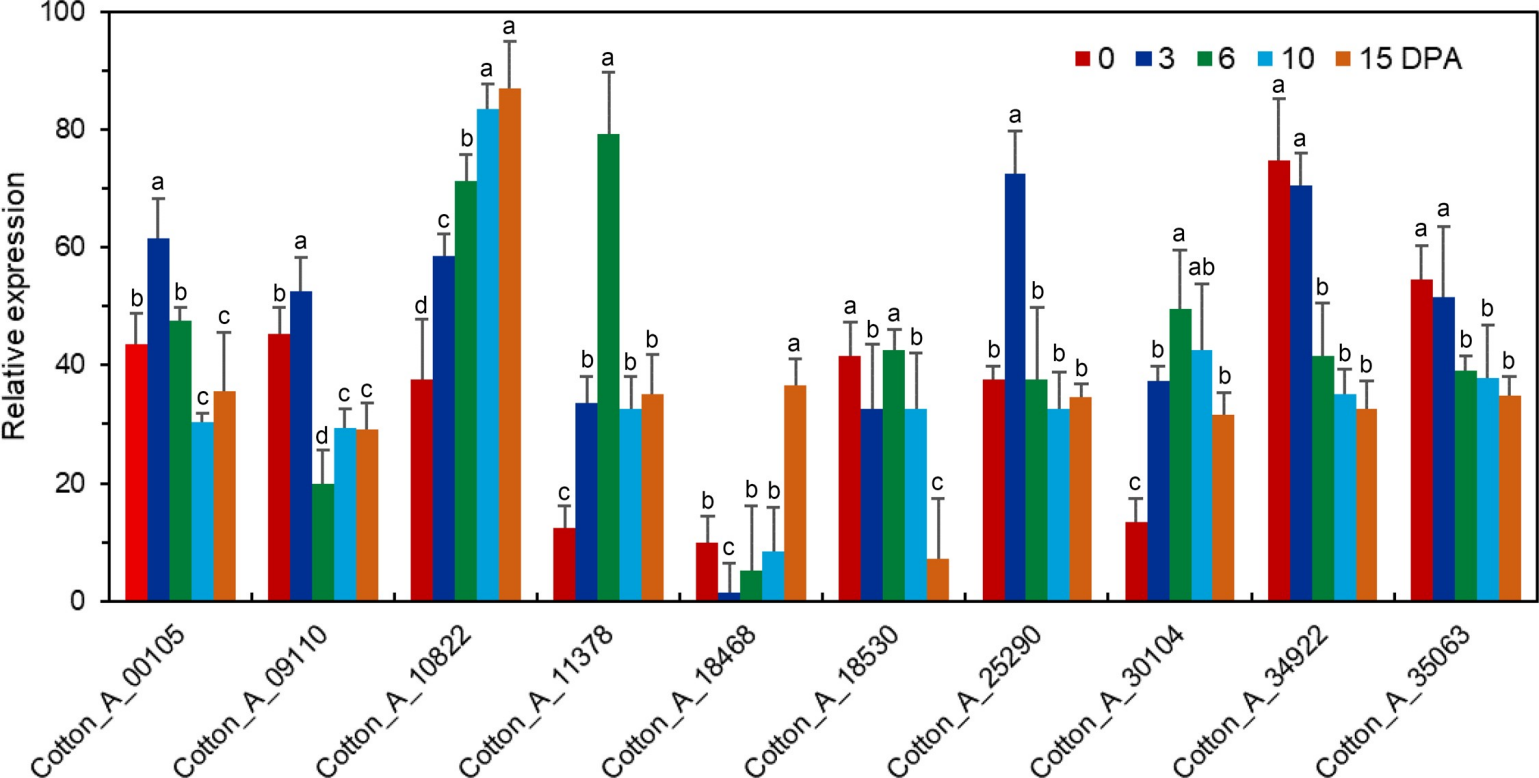
Expression patterns of the selected *RB-GRP* genes in fibers of *G*.*arboreum* during different developmental stages.

**Fig 9:**
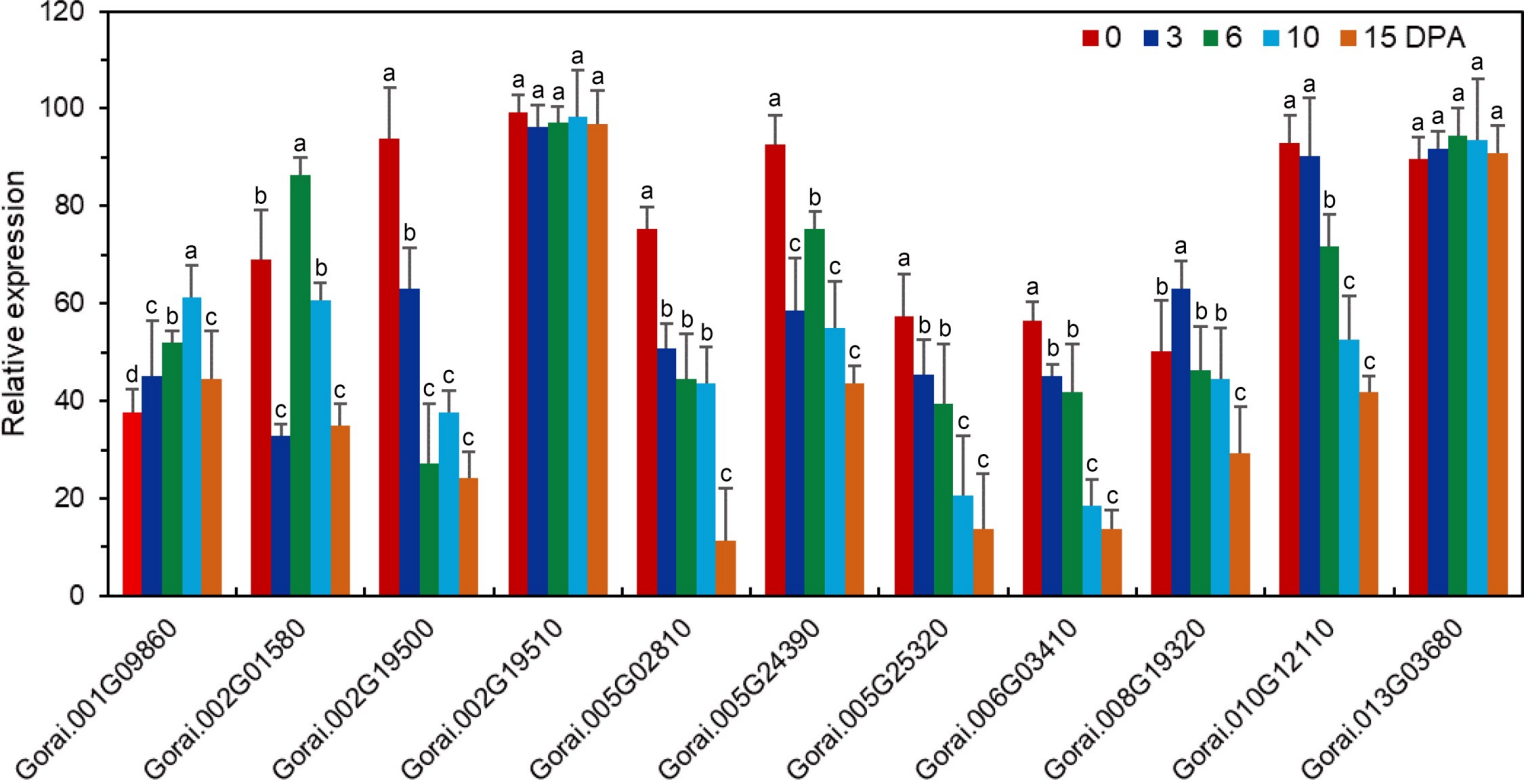
Expression patterns of the selected *RB-GRP* genes in fibers of *G*.*raimondii* during different developmental stages.

**Fig 10:**
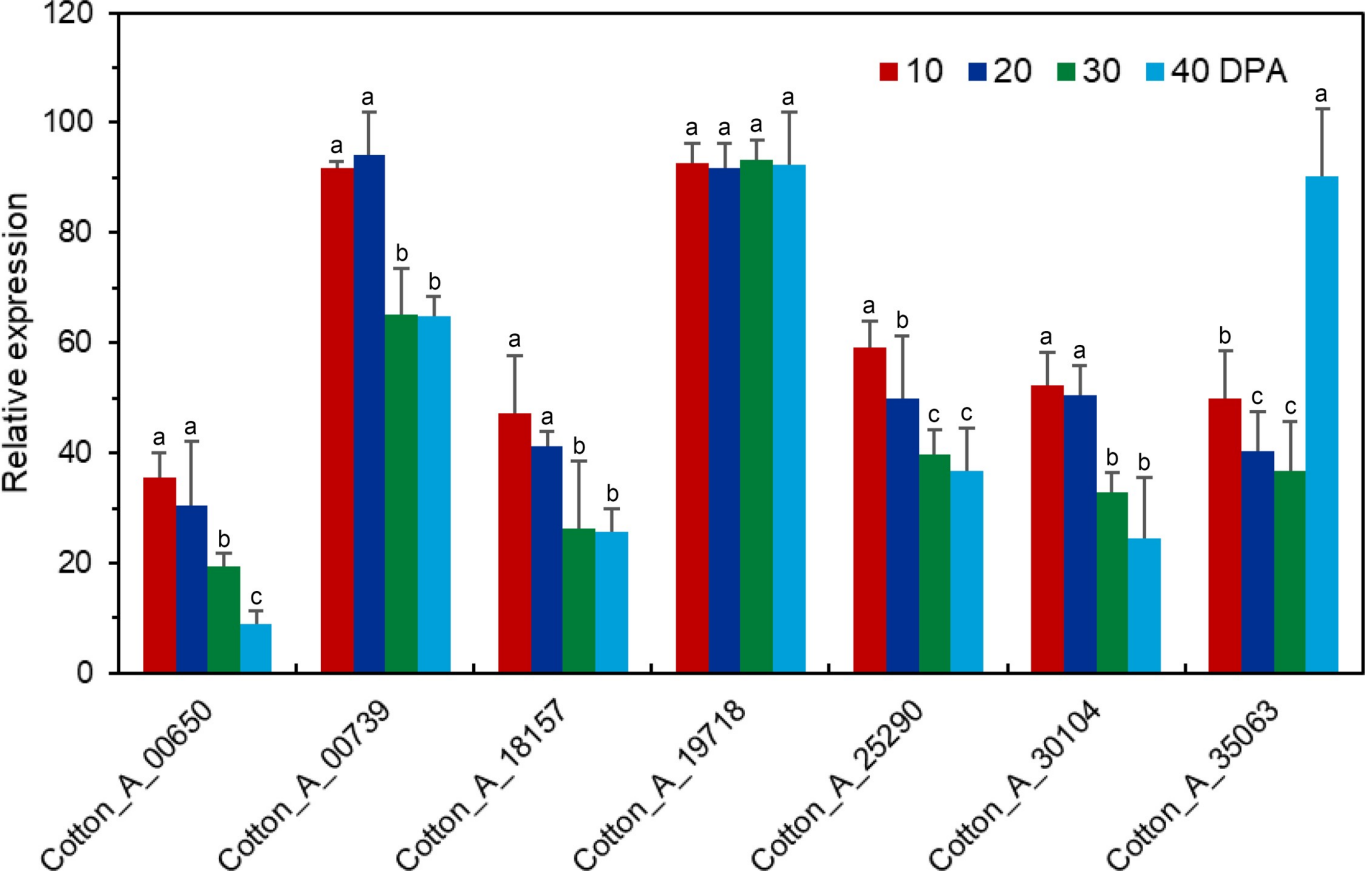
Expression patterns of the selected *RB-GRP* genes in seeds of *G*.*arboreum* during different developmental stages.

**Fig 11:**
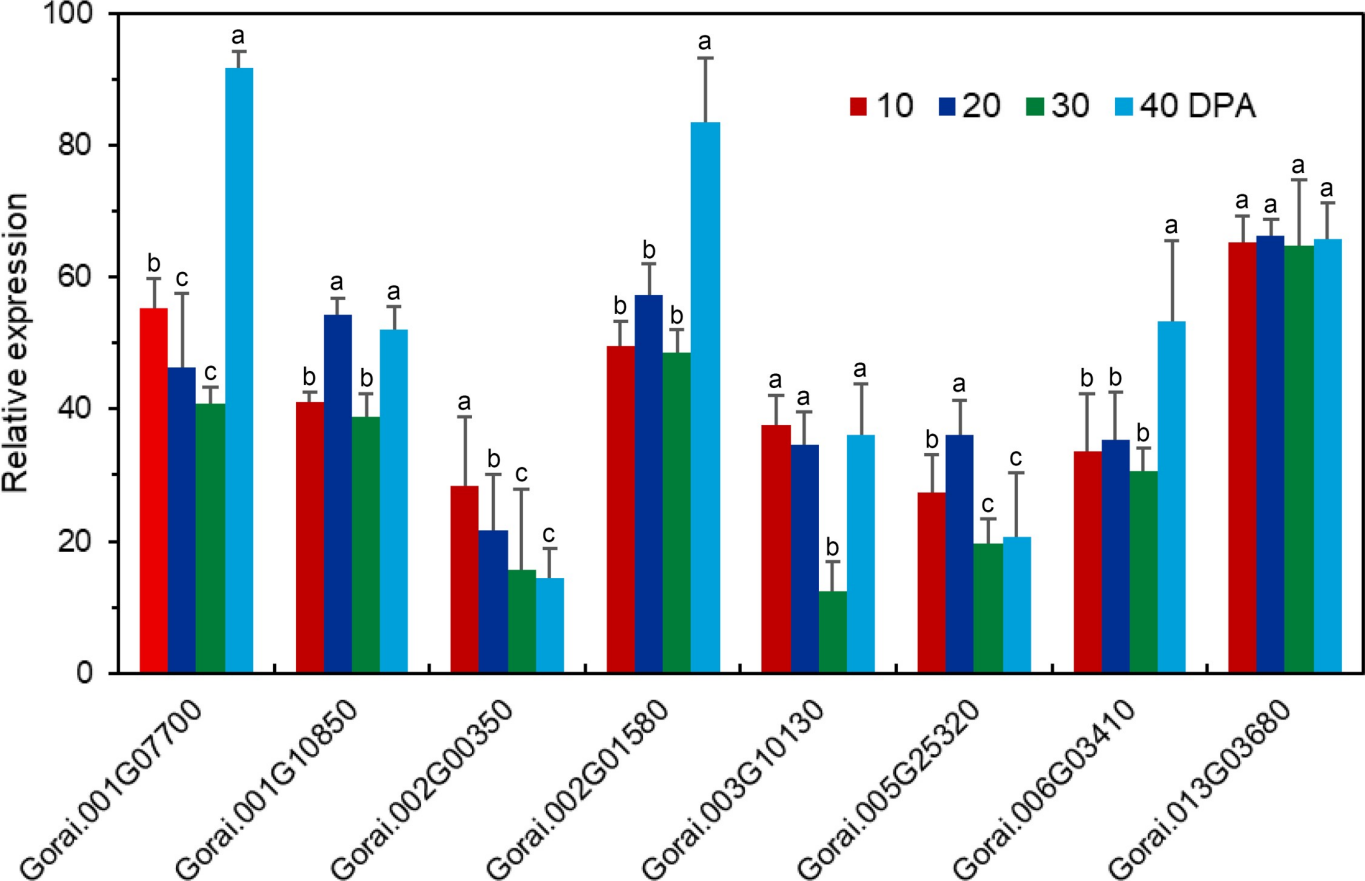
Expression patterns of the selected *RB-GRP* genes in seeds of *G*.*raimondii* during different developmental stages.

We used RNA-seq analysis to compare the gene expression profiles in fiber or seed during different development, the result showed that 10 *GaRB-GRPs* and 11 *GrRB-GRPs* were participated in fiber cell development (Fig 6), 7 *GaRB-GRPs* and 11 *GrRB-GRPs* were then participated in seed development of *G*.*arboreum* and *G*.*raimondii*, respectively (Fig 7). Expressions of above genes during different development were further analyzed by q-PCR. Expression of *Cotton_A_00105*, *Cotton_A_09110*, *Cotton_A_11378*, *Cotton_A_25290*, *Cotton_A_34922* and *Cotton_A_35063* were higher in the earlier development stage of fiber, especially *Cotton_A_34922* and *Cotton_A_35063* had the highest expression level at 0–3 DPA, and decreased with fiber development, while *Cotton_A_10822* presented a gradually increasing trend during 0–15 DPA (Fig 8). Unlike *GaRB-GRPs*, most *GrRB-GRPs* presented stable high expression during 0–3 DPA (Fig 9), which means that *GrRB-GRPs* played an important role in earlier fiber development of *G*.*raimondii*. In addition, while two (*Gorai*.*002G19510* and *Gorai*.*013G03680*) kept high expressions during 0–15 DPA (Fig 9), indicated that they may participated in the initiation and elongation of cotton fiber. There are many *RB-GRP*s differentially expressed during the seed development of cotton (Figs 10 and 11). In *G*.*arboreum*, expressions of most *RB-GRP*s showed a decline trend with seed development of cotton, while *Cotton_A_19718* kept high and stable expression during whole seed developmental stage, and *Cotton_A_35063* showed a dramatic increase at 40 DPA (Fig 10). But in *G*.*raimondii*, only *Gorai*.*013G03680* showed a high stable expression in seed during different developmental stages. Expressions of *Gorai*.*002G07700*, *Gorai*.*002G01580* and *Gorai*.*006G03410* presented transient increase in seed at 40 DPA (Fig 11).

### Expression analysis of *RB-GRPs* between *G*.*arboreum* and *G*.*raimondii* under different stress conditions

**Fig 12:**
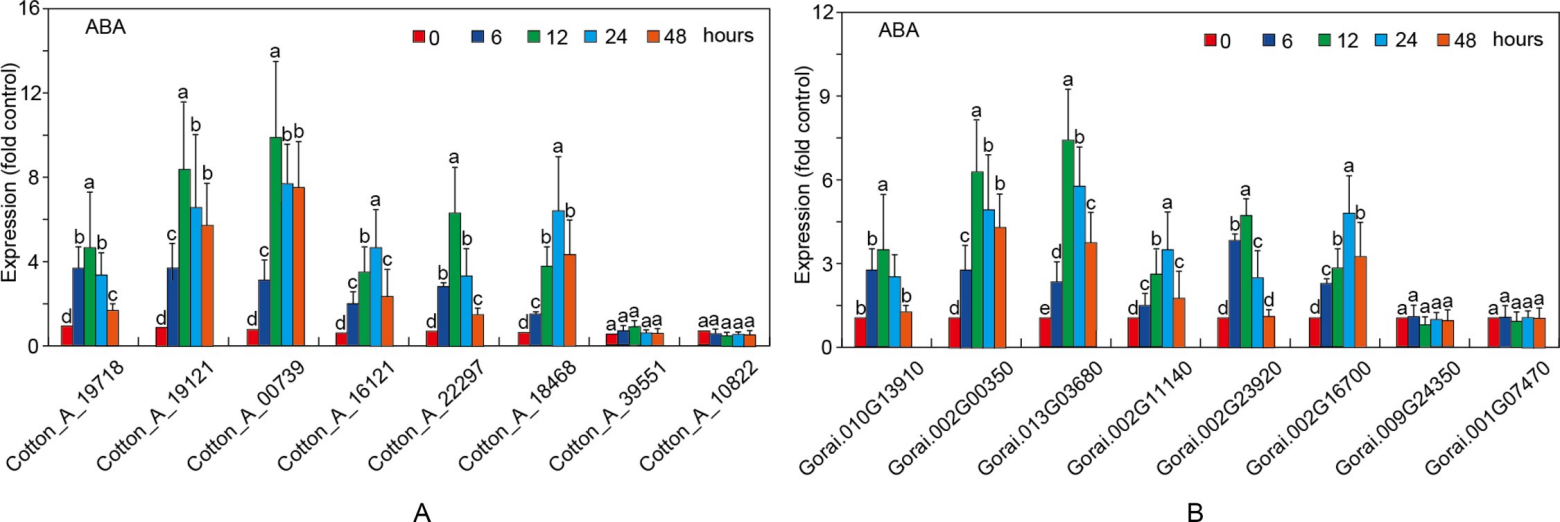
Expression patterns of the selected *RB-GRP* genes in *G*.*arboreum* (A) and *G*.*raimondii* (B) under ABA treatment.

**Fig 13:**
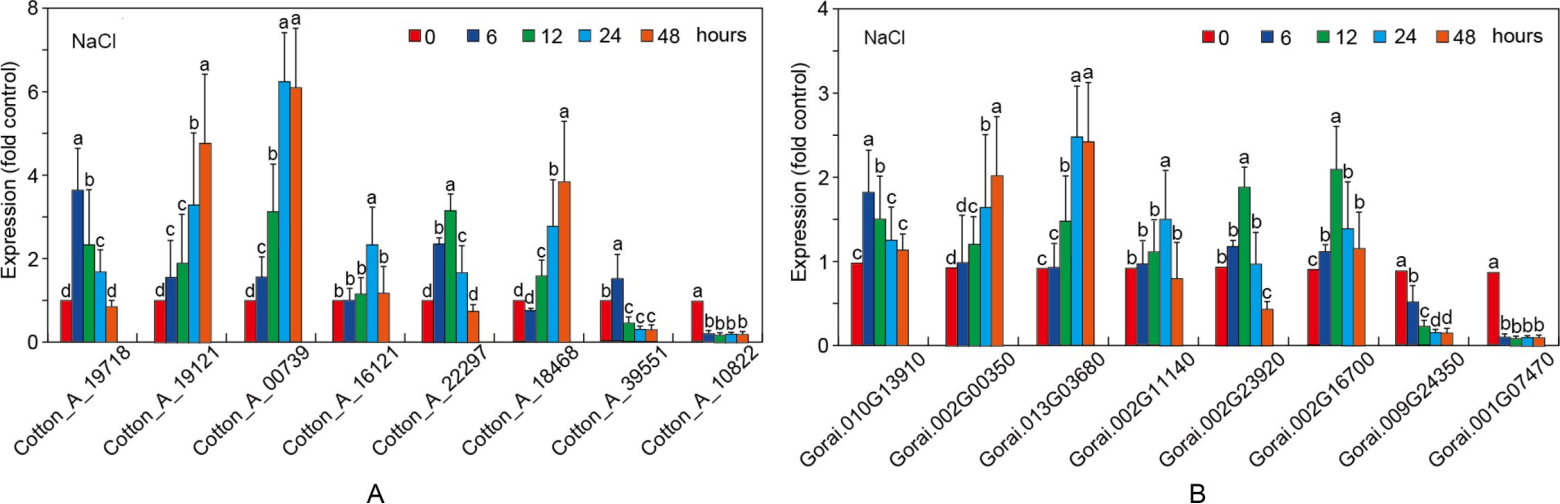
Expression patterns of the selected *RB-GRP* genes in *G*.*arboreum* (A) and *G*.*raimondii* (B) under salt treatment.

**Fig 14:**
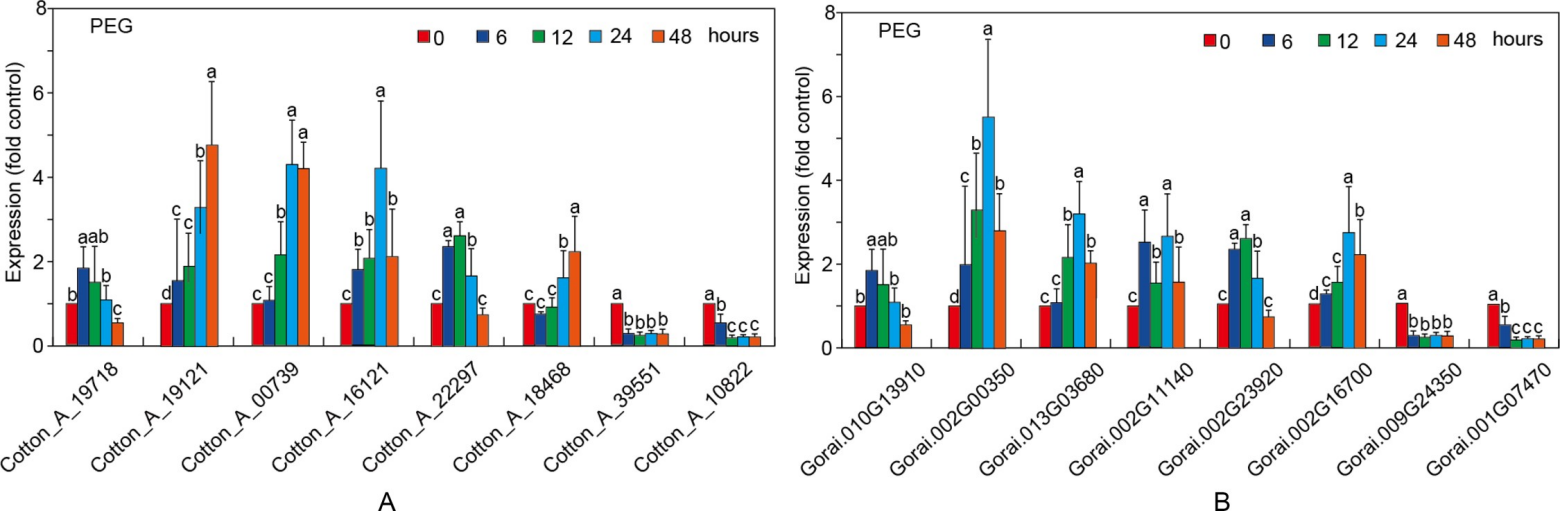
Expression patterns of the selected *RB-GRP* genes in *G*.*arboreum* (A) and *G*.*raimondii* (B) under PEG treatment.

To understand stress responsiveness of *GaRB-GRPs* and *GrRB-GRPs*, eight *GaRB-GRPs* and *GrRB-GRPs* were chosen for expression profile analysis under different stress conditions, ABA, NaCl and PEG. Six genes (*Cotton_A_19718*, *Cotton_A_19121*, *Cotton_A_00739*, *Cotton_A_16121*, *Cotton_A_22297* and *Cotton_A_18468*) in *G*.*arboreum* were upregulated at first then downregulated, while *Cotton_A_39551* and *Cotton_A_10822* had the least changes under the stress of ABA (Fig 12). There were also six genes (*Gorai*.*010G13910*, *Gorai*.*002G00350*, *Gorai*.*013G03680*, *Gorai*.*002G11140*, *Gorai*.*002G23920* and *Gorai*.*002G16700*) in *G*.*raimondii* participated in ABA response, and presented the same trend as stress prolonging, while *Gorai*.*009G24350* and *Gorai*.*001G07470* showed insensitive to ABA stress (Fig 12). As shown in Fig 13, all the eight *RB-GRPs* both in two cotton species responded to salt tress treatment. Expressions of *Cotton_A_19121*, *Cotton_A_000739* and *Cotton_A_18468* were increased, while that of the others upregulated at first then downregulated (expect for *Cotton_A_10822*) (Fig 13). In comparison, *GrRB-GRPs* in *G*.*raimondii* are relative insensitivity to salt stress (Fig 13). Four *GrRB-GRPs* (*Gorai*.*010G13910*, *Gorai*.*002G11140*, *Gorai*.*002G23920* and *Gorai*.*002G16700*) rose at first then decreased, two *GrRB-GRPs* (*Gorai*.*002G00350*, *Gorai*.*013G03680*) increased under salt stress, and expressions of other *GrRB-GRPs* (*Gorai*.*009G24350*, *Gorai*.*001G07470*) showed lower than control (Fig 13). Expressions of *Cotton_A_19121*, *Cotton_A_00739* and *Cotton_A_18468* were up-regulated, while three *GaGB-GRPs* (*Cotton_A_19718*, *Cotton_A_16121* and *Cotton_A_22297*) and five *GrGB-GRPs* (*Gorai*.*010G13910*, *Gorai*.*002G00350*, *Gorai*.*013G03680*, *Gorai*.*002G23920* and *Gorai*.*002G16700*) increased at first then decreased after PEG treatment (Fig 14).

## Discussion

### *RB-GRP* genes in different cotton species

RB-GRP family is characterized by the presence of a glycine-rich domain arranged in (Gly)n-X repeats and a RRM [7]. A number of *RB-GRPs* have been reported in different plants, such as eight in *Arabidopsis* [25] and six in rice [26], which play important roles in plant growth, development, and stress resistance [8, 9]. In our study, 37 *GaRB-GRPs* and 32 *GrRB-GRPs* were identified in genomes of *G*.*arboreum* and *G*.*raimondii*. Conserved protein sequence analysis indicated that the *GaRB-GRPs* and *GrRB-GRPs* could be divided to four subgroups (Class IVa, IVb, IVc and IVd), which was similar to that in other plant species [3]. However, two *GaRB-GRPs* (*Cotton_A_35374* and *Cotton_A_38093*) and one *GrRB-GRP* (*Gorai*.*002G167100*) belong to Class IVb contained one RRM domain and two ZnF_RanBP_**2**_, which is not found in other plant species.

The species phylogenetic tree displayed the value of synonymous substitution/site (Ks) among *G*.*arboreum*, *G*.*raimondii* and *T*.*cacao*, and the divergence time of cotton species can be calculated by Ks values. The result of divergence time was consistent with that the common ancestor of two diploid cotton species have diverged from *T*.*cacao* 18–58 million years ago [19]. To sum up, we could speculated that *RB-GRP* gene in cotton was in an expansion trend after speciation from the *T*.*cacao* lineage.

### Tandem duplication and segmental duplication contributed to the expansion of the *RB-GRP* gene family in *G*.*arboreum* and *G*.*raimondii*

Gene family expansion mainly caused by tandem duplication, segmental duplication and transposition events [7]. Through the analyses of 50 gene families in *Arabidopsis thaliana*, tandem duplication is the most prominent, whereas segmental and transposition events occurred more frequently in other plants [27]. Due to a recent and an ancient whole-genome duplication event occurring in *G*.*arboreum* and *G*.*raimondii* genomes, and the whole-genome duplication event cannot be ruled out as the cause of gene family expansion in *G*.*arboreum* and *G*.*raimondii*. In our study, no orthologous genes in *G*.*raimondii* could be found corresponding to 11 *GaRB-GRPs*, and 8 *GrRB-GRPs* have no corresponding orthologous genes in *G*.*arboreum*, which may result from losing or deleting of orthologous after whole genome duplication event, and the missing genes may not survive the evolutionary selection pressure. Moreover, the two cotton species experienced a retrotransposon insertion event, and the insertion was the fundamental reason for the different size between A and D genome [28], including the losing or deleting of orthologous genes. The number of copies of the orthologous genes between *G*.*arboreum* and *G*.*raimondii* was not increased by the whole genome duplication. Thus, whole genome duplication of cotton did not contribute to expansion of *RB-GRPs* in *G*.*arboreum* and *G*.*raimondii*.

Only two (5.4%) and four (12.5%) tandem duplication genes were found in *RB-GRPs* among *G*.*arboreum* and *G*.*raimondii*, while seven pairs of paralogous *RB-GRP* genes in *G*.*arboreum* and five pairs of paralogous *RB-GRP* genes in *G*.*raimondii* randomly distributing on chromosomes indicated that segmental duplication is predominant in the three duplication events, which was consistent with the expansion of the *ZmRB-GRPs* in maize [7]. Overall, both tandem duplication and segmental duplication contributed to expansion of *RB-GRP* gene family between *G*.*arboreum* and *G*.*raimondii*.

### Role of the *GaRB-GRPs* and *GrRB-GRPs* during fiber and seed development of cotton

Cotton plays a crucial role in human life, the world economy, and scientific research, and the fiber of cotton is an essential raw material for the textile industry [29, 30], and the seed of cotton, a by-product of fiber, is increasingly recognized to have excellent potential as a source of food and biofuel [31]. A large number of genes are believed to be involved in fiber and seed developmental process, such as *WLIM1a*, *Sus* (Sucrose synthase), *GhRDL1* [32, 33, 34]. The tissue-specific expression patterns of the selected *RB-GRP* genes under normal condition reflected that they might play versatile functions in the growth and development of cotton (Dong et al. 2016). Comparing to *G*.*arboreum*, there were more *RBGRPs* involve in the cotton fiber development of *G*.*raimondii*, while more *RBGRPs* differently expressed in seed of *G*.*arboreum*. Although most of the expression patterns between GaRB-GRP encoding genes and GrRB-GRP encoding genes were different in same tissue between *G*.*arboreum* and *G*.*raimondii*, few orthologous genes presented similar expressions between two cotton species, which implied their functional conservation.

### Role of the *GaRB-GRPs* and *GrRB-GRPs* under different stresses tolerance

Most of Arabidopsis, rice and maize *RB-GRPs* were involved in response to stresses [5, 6, 7]. In this study, expressions of 8 *GaRB-GRPs* and 8 *GrRB-GRPs* in two cottons under different stresses were analyzed by qPCR. *Cotton_A_19718*, *Cotton_A_19121*, *Cotton_A_00739*, *Cotton_A_16121*, *Cotton_A_22297*, *Cotton_A_18468* in *G*.*arboreum* and *Gorai*.*010G13910*, *Gorai*.*002G00350*, *Gorai*.*013G03680*, *Gorai*.*002G11140*, *Gorai*.*002G23920*, *Gorai*.*002G16700* in *G*.*raimondii* were significantly responded to ABA, NaCl and PEG response, while *Cotton_A_39551*, *Cotton_A_10822* in *G*.*arboreum* and *Gorai*.*009G24350*, *Gorai*.*001G07470* in *G*.*raimondii* were involved in the early stages of different stress, while showed no obvious significant difference with the prolongation of treatment, which implied that they were involved in rapid response to environmental stress, and other *RB-GRPs* play an important role in stress resistance. The gene expression patterns can provide important clues for gene function [35]. However, their detailed roles in stress responses need to be further studied in future.

## Conclusion

The *RB-GRP* gene family has been extensively studied in model plant species such as *Arabidopsis*, rice and mazie [5, 6, 7], while there has been a lack of systematic analysis of *RB-GRP* family genes in cotton. Here, we identified and compared the *RB-GRP* gene family members between the two cotton species (*G*. *raimondii* and *G*. *arboretum*). The *RB-GRP* genes in cotton likely experienced tandem and segmental duplication events, similar to other species. Most *RB-GRPs* in two cotton species undergone stronger negative selective pressure by evolutionary analysis of *RB-GRP* orthologous genes. qRT-PCR analyses revealed that *RB-GRPs* have crucial functions during fiber and seed development of cotton, and may be involved in ABA, NaCl and PEG response. The results have provided a basis for further assessment of physiological roles of different *RB-GRP* genes in response to stresses in cotton species.

## Supporting information

S1 Fig Phylogenetic tree of RB-GRP genes in *G*. *arboreum*. (TIF)

S2 Fig Phylogenetic tree of RB-GRP genes in *G*. *raimondii*. (TIF)

S3 Fig Comparative analysis of Ka/ks ratio value for RRM-type and CSD-type *RB-GRP* genes between *G*. *arboreum* and *G*. *raimondii*. (TIF)

S1 Table *RB-GRP* gene primer pairs used in the q-PCR experiments. (DOCX)
