# Supplementary figures and images for: Genome-wide comparative analysis of RNA-binding Glycine-rich protein family genes between Gossypium arboreum and Gossypium raimondii
Source: PLoS One. 2019 Jun 26;14(6):e0218938. doi: 10.1371/journal.pone.0218938 (PMC6594650; doi:10.1371/journal.pone.0218938)

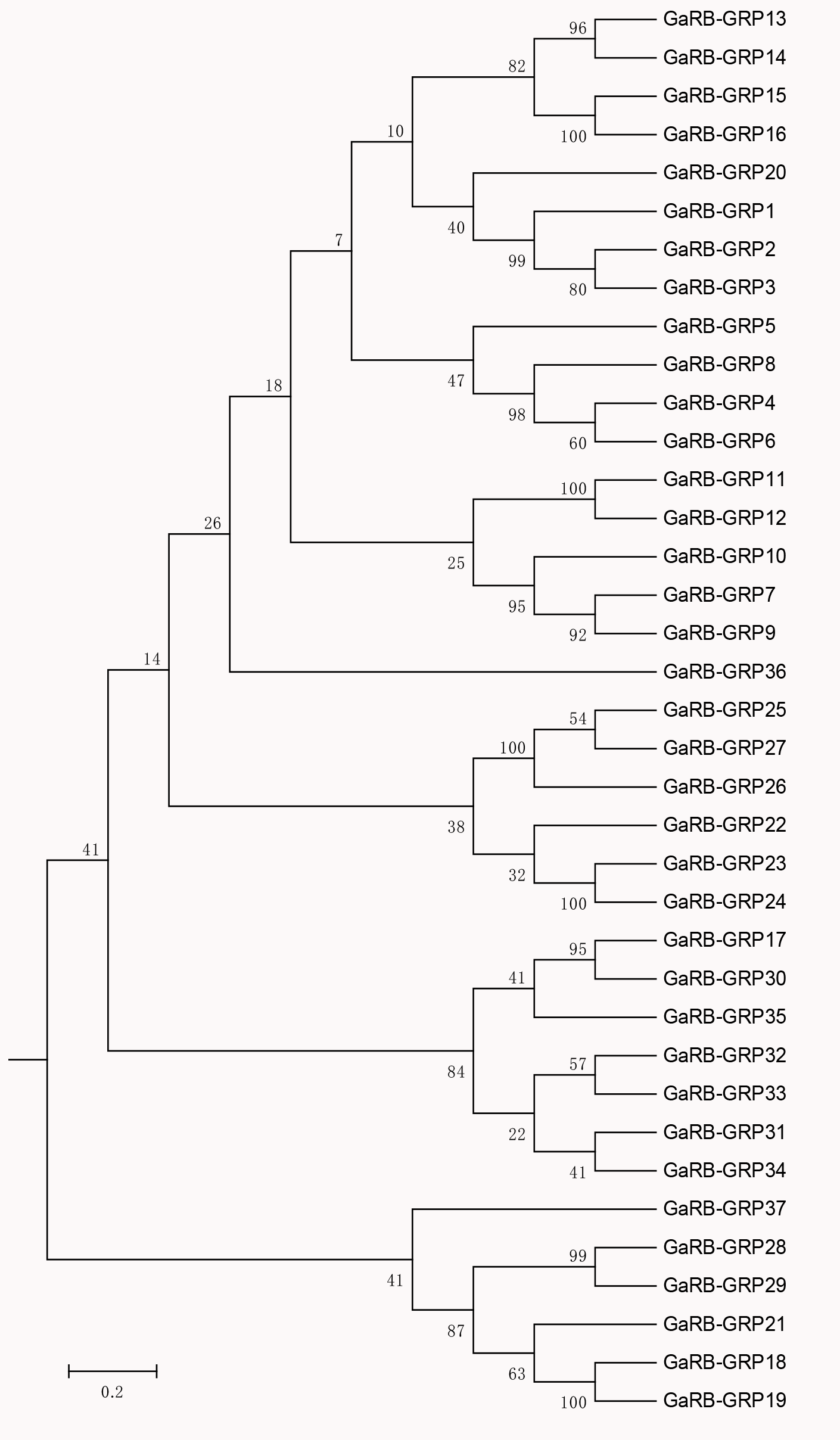

Supplement: S1 Fig — (TIF) [file pone.0218938.s001.tif]

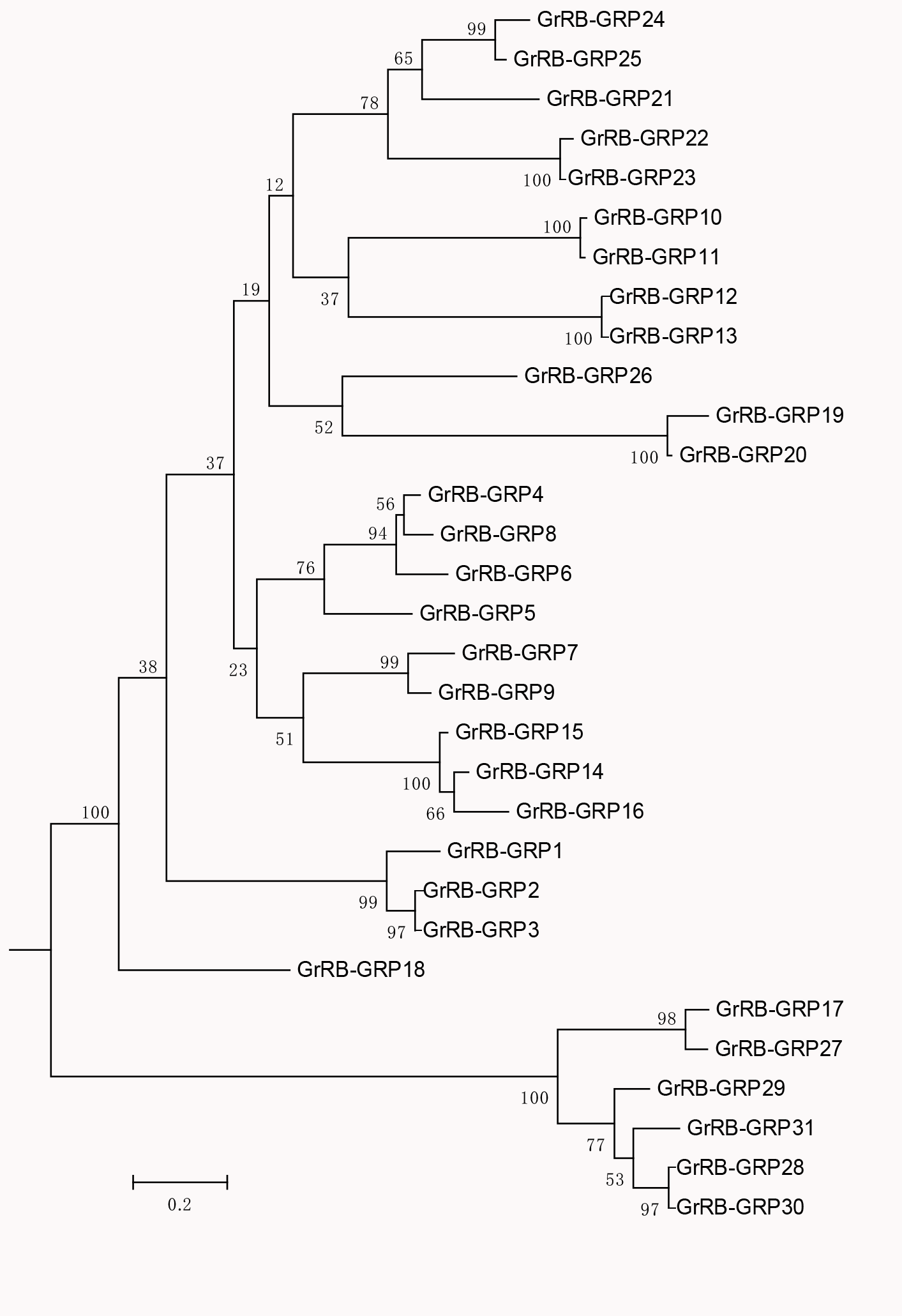

Supplement: S2 Fig — (TIF) [file pone.0218938.s002.tif]

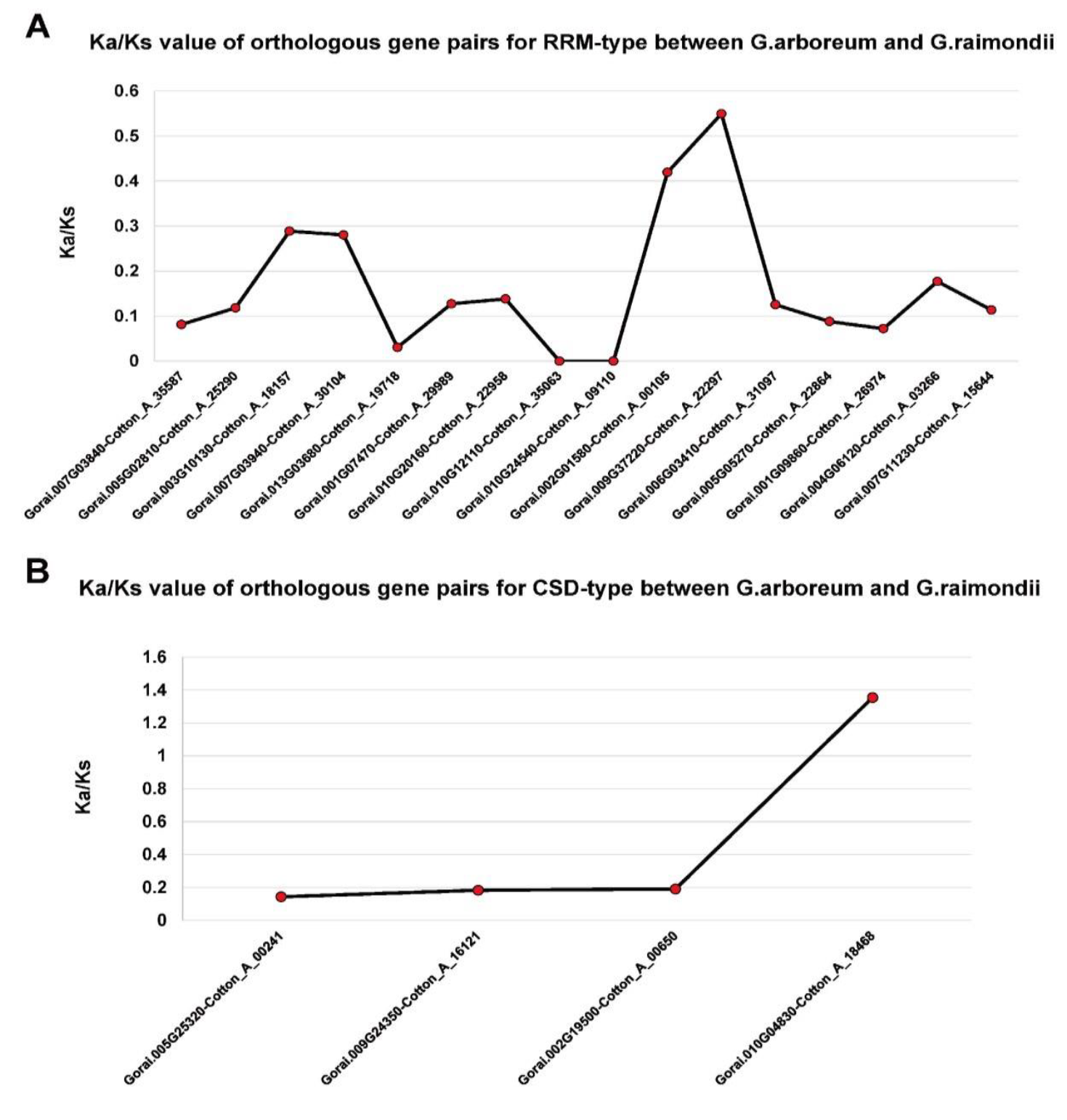

Supplement: S3 Fig — (TIF) [file pone.0218938.s003.tif]
