# Supplementary material for: Genome-wide comparative analysis of RNA-binding Glycine-rich protein family genes between Gossypium arboreum and Gossypium raimondii
Source: PLoS One. 2019 Jun 26;14(6):e0218938. doi: 10.1371/journal.pone.0218938 (PMC6594650; doi:10.1371/journal.pone.0218938)
Supplement: S1 Table — (DOCX) [file pone.0218938.s004.docx]

**S1 Table. *RB-GRP* gene primer pairs used in the q-PCR experiments.**

| **gene ID** | **Forward (5’ to 3’)** | **Reverse (5’ to 3’)** |
| --- | --- | --- |
| Actin | ATCCTCCGTCTTGACCTTG | TGTCCATCAGGCAACTCAT |
| *Cotton_A_00105* | GCGACTGGAACTTGTCAG | ACTCGGGGTGAGTACTCTTG |
| *Cotton_A_00650* | CCATTTACCCGAGCCATTGTT | GTCGGGCCATTTCGAGAACT |
| *Cotton_A_00739* | ACGATCGTGAGACTGGAAGA | GGCGAGATTGTGCTTCGTTG |
| *Cotton_A_09110* | GAAGGGTCCTGAACCTGGTCAT | TGGGCGACCACTTTCCCGATGT |
| *Cotton_A_10822* | TCTTGACGGAAGGAACATTACC | CCCACCTCCATAGCCTCCTTC |
| *Cotton_A_11378* | ACTTTGCCAGGTGGTTACACCCT | GGGCTTTTCCTTCTGCTCTCGT |
| *Cotton_A_16121* | CGGGGAGTGGGTCCTATCCAA | CTTCAACTCGACCAAATCGAGA |
| *Cotton_A_18157* | ACTGAATCGTGAGGACTGGAA | AGTCCAAGGTCCTTGAAGATA |
| *Cotton_A_18468* | GACCGGTGAGAGCTCACTAGCG | AATCGTTTCCGCGGCTCATCTAG |
| *Cotton_A_18530* | CATGCCGAACGGACGAGCTC | CTGAAAGACCTGGCTAAGGCGCC |
| *Cotton_A_18641* | TCAGGTACTGGTGGTCCTCAA | ACAGCATTGTTGGACCATTCTC |
| *Cotton_A_19121* | TCGAACATGCCGAGCTCAAA | GCCAAATCCTCTAGACCTACCC |
| *Cotton_A_19718* | CATAATGATCGTGAGACTGGAAG | AGCGCGATTGGGCTTCGTTG |
| *Cotton_A_22297* | ATACGTCTTGCCACTGATGCT | CTTACACGCGCCAGATCAAG |
| *Cotton_A_25290* | ATCGCATCAACGAACAGGGAC | GTCTAAAGGACGATATTTTCA |
| *Cotton_A_30104* | TCGATGCGACAAGACATCACCA | CGCCTCCTTCGTTCACACACCA |
| *Cotton_A_34922* | AGAACTAGCACATCAAGAG | TTGCACCACCACTGATGCCCA |
| *Cotton_A_35063* | TCGACATCACCGCCTTACACA | GCTTTGATCATCACTTATGGCTT |
| *Gorai.001G07470* | TTCTGCTCCCACACCGAAGA | TCTTTGCAGTTTTCCCAGTAACA |
| *Gorai.001G07700* | TGGCTTTGTCACTTACACAA | TCCATAATTTCCAGAAGTGGGG |
| *Gorai.001G09860* | GGGATTCCAACATCAGTTACT | ACCTCCAAAAGACCTATAAGG |
| *Gorai.001G10850* | AACCGCTCCGCACCCTATACCG | GGCTTCCCATCAAGCTGAAC |
| *Gorai.002G00350* | GCAATACATCGACGGACGAGA | TGGCTCACAAGAACTAGCCC |
| *Gorai.002G11140* | TTAGGCAAAATGGGCAAACCC | GTCACCGAATCCAGAGAATGC |
| *Gorai.002G01580* | CTTCAGAATCCGAAGATGAAC | AAATCTCTCCTTTCTTAACAG |
| *Gorai.002G16700* | GATCATCTCGACGATCCAAGC | CCTGGTCTTTGGTTCACGTT |
| *Gorai.002G19500* | GACCAAAAGGGTTATGGCTT | CCGGCGCCTCCACCACCAC |
| *Gorai.002G19510* | GTGGTTCGATGACCAAAAGG | CGCCTCCACCACCACCACCAC |
| *Gorai.002G23920* | GACGGCGGCGACGATTTG | TGGGACCAGTGACCTCAACAG |
| *Gorai.003G10130* | TCTCCATGGCCGTCAAGTAA | GTTGATCACCACCCCCAAAT |
| *Gorai.005G02810* | ATGGGCAAACCCCAATGAT | GGGCCGCTAGGTCTTTCATT |
| *Gorai.005G24390* | TAACGATCGTGAGACTGGAAG | CTCCATATCCACCTTCACGGC |
| *Gorai.005G25320* | GTTGGCCGGAACGGTGAAGTG | CCCCGTATCTTCCACCGCCGC |
| *Gorai.006G03410* | CCTCCATCCAAACGATATAAT | CCTCCATAGCGACCCGAGTATC |
| *Gorai.008G19320* | TCTCGTGGATTCGGATTTGTC | ATCACTACCACCACCATAACG |
| *Gorai.009G24350* | GTAACATCAACTGGGCTAAGCG | CGACGTCGCCTTGTTTCCTC |
| *Gorai.010G12110* | TCGGAGCGAGATCTTGAAGACG | GATATCTAGGGCTGCGGCTGCG |
| *Gorai.010G13910* | CTGTTGGGATGCCATCTGGG | AGGTGGTCCGTTGGGATACA |
| *Gorai.013G03680* | CACGTGGTGGTTCTGGAAGAC | GACTTGCTGTAGTTACGCCCA |
